# Catalytic System for Cross-Coupling of Heteroaryl Iodides with a Nitronyl Nitroxide Gold Derivative at Room Temperature

**DOI:** 10.3390/molecules28227661

**Published:** 2023-11-19

**Authors:** Igor Zayakin, Galina Romanenko, Irina Bagryanskaya, Bogdan Ugrak, Matvey Fedin, Evgeny Tretyakov

**Affiliations:** 1 N. D. Zelinsky Institute of Organic Chemistry, Leninsky Ave. 47, Moscow 119991, Russia; zavnsc3@gmail.com (I.Z.); ugrak@ioc.ac.ru (B.U.); 2 International Tomography Center, Institutskaya Str. 3a, Novosibirsk 630090, Russia; romanenko@tomo.nsc.ru; 3 N. N. Vorozhtsov Institute of Organic Chemistry, 9 Ac. Lavrentiev Avenue, Novosibirsk 630090, Russia; bagryan@nioch.nsc.ru

**Keywords:** nitronyl nitroxides, organic gold compounds, cross-coupling, stable radicals

## Abstract

A simple and highly effective methodology for the cross-coupling of heteroaryl iodides with NN–AuPPh_3_ at room temperature is reported. The protocol is based on a novel catalytic system consisting of Pd_2_(dba)_3_·CHCl_3_ and the phosphine ligand ^Me^CgPPh having an adamantane-like framework. The present protocol was found to be well compatible with various heteroaryl iodides, thus opening new horizons in directed synthesis of functionalized nitronyl nitroxides and high-spin molecules.

## 1. Introduction

**Scheme 1:**
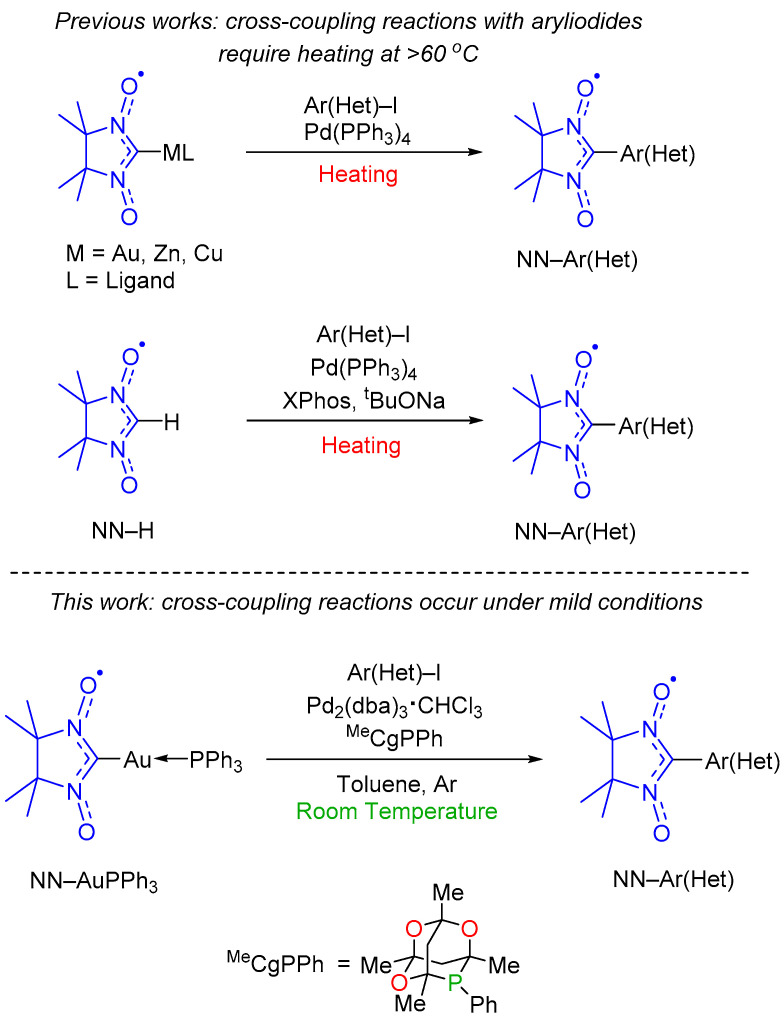
Pd(0)-catalyzed cross-coupling of aryl(heteroaryl)iodides with metal–nitronyl-nitroxide derivatives.

Organic open-shell molecules have attracted considerable attention in different fields of chemistry, materials science, and medicine [1,2,3,4,5]. In the past few decades, various materials and single-molecule systems with fascinating properties based on open-shell molecules have been prepared [6,7,8,9,10]. The use of substituted nitronyl nitroxide radicals (NN-Rs)—either as paramagnetic tectons of pure organic materials or in combination with metal ions in the engineering of heterospin complexes—as sources of a stable spin has played a major role in the development of the field of molecular magnetism [11,12,13]. The widespread use of nitronyl nitroxides, primarily in the area of molecular design of magnets, has contributed to advancements in the chemistry of this class of compounds. An important step was the advent of methods to obtain functionally substituted nitronyl nitroxides using Pd(0)-catalyzed cross-coupling reactions of aryliodides [14,15] or arylbromides [16] with nitronyl nitroxide organometallic derivatives (Scheme 1). It is the cross-coupling reaction that has paved the way to high-spin organic compounds with strong intramolecular ferromagnetic interactions, e.g., verdazyl- [17,18] or triazinyl-substituted nitronyl nitroxides [19] and air-stable magnetically active graphene nanoribbons [12]. These results provide valuable insights and motivation for further progress in the field of cross-coupling methodology applied to nitronyl nitroxide radicals.

Most of the described cross-coupling procedures include the interaction of diamagnetic iodo-substituted aromatic compounds with the triphenylphosphine gold(I)−nitronyl nitroxide complex (NN–AuPPh_3_) in the presence of Pd(PPh_3_)_4_ as a catalyst and require the heating of the reaction mixture at 60–65 °C [20,21]. Rajca and coworkers showed that the cross-coupling of iodo- and diiodo-substituted Blatter radicals with NN–AuPPh_3_ can be carried out at room temperature using a highly reactive Pd(0) catalyst, Pd[(*t*-Bu)_3_P]_2_ [19]. Nevertheless, even with this catalyst, yields of the corresponding di- and triradicals failed to exceed 44%. Recently, we prepared nitronyl nitroxide organogold derivatives NN–AuL (L = XPhos, ^Me^CgPPh, or TTMPP) in a toluene solution (up to 100 °C) and found that the derivatives possessed enhanced thermal stability compared to NN–AuPPh_3_ (decomposing at ~75 °C or 65 °C in toluene and tetrahydrofuran, respectively) [16]. The higher thermal stability of NN–AuL allows for the cross-coupling reactions to be conducted at elevated temperatures when even electron-rich aryl bromides react with NN–AuL in the presence of readily available Pd(PPh_3_)_4_, thereby giving substituted nitronyl nitroxides. Having studied the problem of cross-coupling of metalloorganic derivatives of nitronyl nitroxide with bromoarenes, we focused on the next challenge—the development of highly active catalytic systems enabling cross-coupling of aryliodides with NN–AuPPh_3_ at room temperature. The use of such mild conditions is of particular importance in the case of obtaining high-spin systems, when it is necessary to introduce many nitronyl nitroxide groups into the substrate. As it is known, at elevated temperatures and under cross-coupling reaction conditions, nitronyl nitroxides can undergo deoxygenation and form iminonitroxides, leading to contamination of the target product with impurities that are difficult to separate. This work addresses this long-standing problem that complicates the synthesis of multi-spin systems. As a result of systematic research, we designed a simple and effective catalytic system for the cross-coupling of aryl(hetaryl)iodides with NN–AuPPh_3_ under mild conditions.

## 2. Results and Discussion

The logic of the study was as follows. Initially, *p*-bromoanisole was specifically chosen as an aryl halide because it has low reactivity compared to aryliodides, a fact which is convenient for finding the most active catalytic system. The most commonly used reagent, the NN–AuPPh_3_ gold complex, was selected as the second cross-coupling component. The Pd_2_(dba)_3_·CHCl_3_ complex served as a widely known source of palladium. All reaction conditions were the same except for the fact that various phosphine ligands were added to the reaction mixture. Durations of the reaction until complete disappearance of NN–AuPPh_3_ and yields are summarized in Table 1. 

One can see that the cross-coupling reactions either in the presence of 40 mol% of a bulky electron-rich phosphine [SPhos, TTMPP, rac–BINAP (20 mol%), tris(4-methoxyphenyl)phosphine, tris(4-tolyl)phosphine, or dppf (10 mol%); entries 12–17 in Table 1] or in the absence of any phosphine ligand (entry 1) gave the desired product only in trace amounts. Similar results were obtained with triphenylphosphine or fluorinated triphenylphosphines (entries 18–21). On the other hand, the addition of 20 mol% of dppf rather than 10 mol% raised the yield up to 47%, which is comparable to that obtained in the case of XPhos or *tert*-Bu_3_P (entries 4, 6, and 8, respectively, in Table 1). A significant increase in the yields of cross-coupling products was achieved via the use of phosphine ligands having an adamantane-like framework, ^Me^CgP–R (entries 2, 3, 5, 7, 9, 10, and 11). Notably, the substituent in the backbone of these phosphines ^Me^CgP–R also substantially affected the yields of the cross-coupling product, and the highest yield was reproducibly attained with phosphine ^Me^CgPPh (entry 2, yield 72%).

The next step in the work was a comparative study of various catalytic systems, including the most effective catalytic system [Pd_2_(dba)_3_·CHCl_3_/^Me^CgPPh], in the reaction of *p*-iodoanisole with NN–AuPPh_3_ at room temperature. As expected, despite the decrease in temperature, the use of the more active *p*-iodoanisole instead of the bromo derivative reduced the difference in the activity of catalytic systems with different phosphine ligands (Table 2). A striking example is the result of reactions conducted in the presence of phosphine (4-Tol)_3_P, where in the case of *p*-bromoanisole the traces of the target product were obtained, while with *p*-iodoanisole the yield of the cross-coupling product was 68%. Nevertheless, the highest yield was also obtained when the reaction was conducted in the presence of phosphine ^Me^CgPPh.

The obtained results clearly reveal the influence of the phosphine ligand on the catalytic system for cross-coupling of *p*-bromo- or *p*-iodoanizole with NN–AuPPh_3_. It means that the phosphine structure has a significant impact on so-called “cocktails” of catalysts and on all dynamic processes in this catalytic system including the formation of nanoparticles and leaching [22,23].

Then, we applied the most effective catalytic system [Pd_2_(dba)_3_·CHCl_3_/^Me^CgPPh] to the cross-coupling of various heteroaromatic iodides with NN–AuPPh_3_. The choice of heterocyclic iodides is due to the fact that they are in demand in various fields of research, primarily for the creation of high-spin systems and in the molecular design of magnets. First, we examined various heteroaryl iodides and observed that the cross-coupling reactions proceeded at room temperature, and the reaction showed high tolerance of the functional group (Table 3). In most cases, the resulting products **1**–**14** were isolated in good-to-excellent yields. Among the examined iodides, the most active substrates were thiophene derivatives (entries 1 and 2, Table 3); for example, in the case of 2,5-diiodothiophene, the yield of diradical **1** reached 92%. Moreover, various iodopyrazole derivatives showed high activity in the cross-coupling reaction, e.g., 4-iodo-1-methylpyrazole, 5-iodo-1-difluoromethylpyrazole, and 1,4-bis(4-iodopyrazole-1-yl)benzene (entries 5, 6, and 8, respectively). Good-to-excellent yields were obtained with iodo-derivatives of fluorine-substituted benzothiazoles (entries 3 and 4, respectively), 4-iodopyridine and a derivative of 5-iodopyrimidine (entries 13 and 14, respectively). Derivatives of 4-iodoisoxazole (entries 11 and 12, respectively) and 4-iodo-1-methyl-3-nitropyrazole (entry 7) manifested moderate reactivity, which is attributable to a steric influence of an adjacent alkyl or nitro group. The lowest yield was attained with 3-iodo-1-methylpyrazole (entry 10); this result can be explained by partial blocking of the active catalytic centers by the nitrogen atoms of the pyrazole ring. This supposition is supported by the finding that replacing the Me group with a bulky *iso*-Pr group led to an increase in the yield of the reaction product (entry 9). Last but not least, using the reaction of 5-ethyl-4-iodoisoxazole with NN–AuPPh_3_ as an example, we assessed the influence of a molar concentration of phosphine ^Me^CgPPh on the yield of cross-coupling product **11**. It was observed that a twofold decrease or increase in the molar amount of phosphine ^Me^CgPPh lowered the yield of the reaction product (entry 11).

The molecular and crystal structures of novel nitronyl nitroxides **2**–**4**, **7**–**9**, **11**, and **13** were solved by single-crystal X-ray diffraction analysis (crystallographic data, molecular structures, and fragments of packings are provided in the Supporting Information). All obtained nitronyl nitroxide radicals were investigated using Electron Paramagnetic Resonance (EPR) spectroscopy. The observed spectra were typical of nitronyl nitroxides with two equivalent ^14^N hyperfine interaction constants (~0.72–0.76 mT) and g-values (g~2.007) characteristic of such radicals (more details in the Supporting Information).

**Scheme 2:**
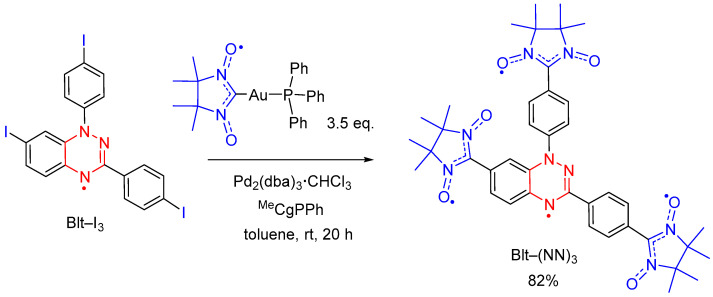
Synthesis of tetraradical Blt–(NN)_3_.

The effectiveness of the proposed catalytic system has been proven by the following two experiments. When using the catalytic system [Pd_2_(dba)_3_·CHCl_3_/^Me^CgPPh], the yield of nitronyl nitroxide **2** was 91%, whereas in the presence of Pd(PPh_3_)_4_ as a catalyst at the same temperature and reaction duration the yield of paramagnetic **2** did not exceed 24%. An even more striking example indicating the promise of using the new catalytic system in the synthesis of multi-spin systems is the preparation of a Blt–(NN)_3_ tetraradical in a yield of 82% (Scheme 2) [24]. Note that the high efficiency of the catalytic system [Pd_2_(dba)_3_·CHCl_3_/^Me^CgPPh] made it possible to obtain the tetraradical Blt–(NN)_3_ in its pure form and to reveal its inherent magnetic-structural correlations.

## 3. Materials and Methods

### 3.1. General Procedures and Materials

(Nitronyl nitroxide-2-ide)(triphenylphosphine)gold (NN–AuPPh_3_) [25], Pd_2_(dba)_3_·CHCl_3_ [26], 1,3,5,7-tetramethyl-8-aryl-2,4,6-trioxa-8-phosphaadamantane (^Me^CgP–Ar) [27], ^Me^CgPCH_2_OH [28], and ^Me^CgPBr [29] were synthesized according to reported procedures. All other organic reagents were purchased from commercial suppliers (Sigma-Aldrich, Darmstadt, Germany; TCI Chemicals, Chennai, India) and were used as received. Toluene was distilled under an argon stream and kept in an argon atmosphere. Other solvents were of reagent quality and used without additional purification. The reactions were monitored by thin-layer chromatography on silica gel 60 F254 aluminium sheets from Merck (Darmstadt, Germany). The chromatography was carried out using silica gel (0.050–0.160 mm) for column chromatography. The yields are given for pure substances obtained after recrystallization. Melting points were measured by means of Stuart melting point apparatus SMP 30; solvents used for recrystallization are indicated after the melting points.

NMR spectra of samples in CDCl_3_ were recorded on a Bruker AV300 (Bruker Corporation, Billerica, MA, USA) spectrometer. In ^1^H NMR spectra (300 MHz), signals were presented in reference to the deuterated solvent peaks; in case of ^31^P NMR spectra (121 MHz), H_3_PO_4_ was acquired as an internal reference. Fourier transform infrared (FTIR) spectra were registered using a BRUKER Vertex-70 FTIR (Bruker Corporation, Billerica, MA, USA) spectrometer.

EPR measurements were conducted on a Jeol JES-FA200 X-band (JEOL Ltd., Tokyo, Japan) spectrometer using a Jeol X-Band Microwave Unit (Tokyo, Japan) (9.8 GHz) at 290 K in dilute (down to approximately 10^−5^ M) toluene solutions degassed by argon bubbling. The spectra were recorded during one slow (~1 h) scan with a modulation of 0.2 mT at 100 kHz and a power of 4 mW. Isotropic g-factor values were measured experimentally using MgO doped with Mn(II) ions as a standard placed in the resonator simultaneously with the test solution. The spectra were simulated using the EasySpin toolbox for Matlab [30]. Figure S9 shows the experimental and simulated EPR spectra of the studied nitronyl nitroxides.

### 3.2. Synthesis of Phosphines ^Me^CgP–R

#### 3.2.1. Synthesis of 1,3,5,7-Tetramethyl-8-(2′,4′,6′-trimethoxyphenyl)-2,4,6-trioxa-8-phosphaadamantane

*n*-BuLi (2.5 mL, 2.5 M solution in THF, 1.05 equiv.) was added to the solution of 1,3,5-trimethoxybenzene (1.0 g, 5.95 mmol, 1.0 equiv.) in dry THF (12 mL) at −30 °C. It was stirred for 3 h at room temperature to produce white suspension. Then, the solution of ^Me^CgPBr (1.75 g, 5.95 mmol, 1.0 equiv.) in THF (20 mL) was added at −30 °C dropwise. After stirring overnight at room temperature, NH_4_Cl (saturated aqueous solution, 100 mL) was added and it was extracted with CH_2_Cl_2_ (3 × 25 mL) and dried with Na_2_SO_4_. After solvent evaporation, the reaction mixture was purified by column chromatography (2.5 cm × 8 cm) with hexane/Et_2_O (5:4 *v*/*v*). The product was obtained as an air-stable white powder. (1.36 g, 3.56 mmol, 60%). ^1^H NMR (300 MHz, CDCl_3_) δ 6.12 (s, 2H), 3.85 (s, 3H), 3.81 (s, 6H), 2.36 (dd, *J* = 13.2, 7.8 Hz, 1H), 2.02–1.79 (m, 2H), 1.56 (dd, *J* = 12.9, 3.9 Hz, 1H), 1.46 (d, *J* = 13.1 Hz, 3H), 1.40 (d, *J* = 7.8 Hz, 6H), 1.26 (d, *J* = 11.1 Hz, 3H). ^31^P NMR (121 MHz, CDCl_3_) δ −29.40. FTIR (KBr, cm^−1^) 432, 474, 521, 567, 587, 640, 678, 689, 728, 804, 826, 851, 893, 915, 950, 978, 1036, 1084, 1129, 1153, 1179, 1208, 1225, 1263, 1289 1338, 1378, 1400, 1456, 1515, 1575, 1594, 1716, 1782, 1952, 2030, 2072, 2107, 2157, 2252, 2287, 2557, 2737, 2837, 2856, 2913, 2935, 2966, 2988, 3442. Elemental analysis: found (%) C 58.77, H 6.88; calculated for C_19_H_27_O_6_P: C 59.68, H 7.12.

#### 3.2.2. Synthesis of 1,3,5,7-Tetramethyl-8-(2′,4′,6′-trimethoxy-[1′,1″-biphenyl]-2-yl)-2,4,6-trioxa-8-phosphaadamantane

*n*-BuLi (0.650 mL, 2.5 M solution in THF, 1.05 equiv.) was added to the solution of 2′-bromo-2,4,6-trimethoxy-1,1′-biphenyl (0.500 g, 1.55 mmol, 1.0 equiv.) in dry THF (10 mL) at −78 °C. It was stirred for 3 h at room temperature to produce white suspension. Then, the solution of ^Me^CgPBr (0.460 g, 1.55 mmol, 1.0 equiv.) in THF (5 mL) was added at −78 °C dropwise. After stirring overnight at room temperature, the solvent was evaporated under reduced pressure. The crude material was dissolved in CHCl_3_ and subjected to column chromatography with hexane/CHCl_3_ (from 1:1 to 0:1 *v*/*v*). The product was obtained as an air-stable white powder (0.251 g, 0.55 mmol, 35%). ^1^H NMR (300 MHz, CDCl_3_) δ 8.31 (d, *J* = 7.8 Hz, 1H), 7.46–7.31 (m, 2H), 7.21–7.15 (m, 1H), 6.19 (s, 2H), 3.88 (s, 3H), 3.72 (s, 3H), 3.67 (s, 3H), 2.07–1.82 (m, 3H), 1.62 (s, 1H), 1.47 (t, *J* = 6.6 Hz, 6H), 1.36 (s, 3H), 1.00 (d, *J* = 12.2 Hz, 3H). ^31^P NMR (121 MHz, CDCl_3_) δ −34.40. FTIR (KBr, cm^−1^) 415, 436, 453, 470, 506, 572, 586, 632, 688, 754, 783, 800, 828, 853, 894, 948, 978, 1002, 1039, 1085, 1130, 1158, 1208, 1222, 1264, 1336, 1375, 1391, 1412, 1436, 1457, 1504, 1586, 1611, 1693, 1728, 2834, 2879, 2934, 2955, 2987, 3057, 3456. Elemental analysis found (%) C 64.17, H 7.02; calculated for C_25_H_31_O_6_P: C 65.49, H 6.82.

### 3.3. General Procedure for Cross-Coupling Catalytic Optimization with p-Bromoanisole

NN–AuPPh_3_ (0.030 g, 0.049 mmol, 1.0 equiv.), Pd_2_(dba)_3_·CHCl_3_ (0.0050 g, 0.0049 mmol, 0.1 equiv.), and the corresponding phosphine (0.020 mmol, 0.4 equiv.) were dissolved in toluene (1 mL) under an argon atmosphere, then *p*-bromoanisole (0.055 mmol, 1.1 equiv.) was added. The reaction mixture was heated at 70 °C for the reported time (see Table 1). After cooling to room temperature, the reaction solution was loaded onto a SiO_2_ column packed with hexane. The cross-coupling product was eluted with hexane/EtOAc (3:1) and recrystallized from CH_2_Cl_2_/hexane. The results are summarized in Table 1 (see Section 2).

### 3.4. General Procedure for Cross-Coupling Catalytic Optimization with p-Iodoanisole

NN–AuPPh_3_ (0.030 g, 0.049 mmol, 1.0 equiv.), Pd_2_(dba)_3_·CHCl_3_ (0.0050 g, 0.0049 mmol, 0.1 equiv.), and the corresponding phosphine (0.020 mmol, 0.4 equiv.) were dissolved in toluene (1 mL) under an argon atmosphere, then *p*-iodoanisole (0.055 mmol, 1.1 equiv.) was added. The reaction mixture was stirred at room temperature for the reported time (see Table 2). Then, the reaction solution was loaded onto a SiO_2_ column packed with hexane. The cross-coupling product was eluted with hexane/EtOAc (3:1) and recrystallized from CH_2_Cl_2_/hexane. The results are summarized in Table 2 (see Section 2).

### 3.5. General Cross-Coupling Procedure

NN–AuPPh_3_ (1.0 equiv.), Het–I (1.0 equiv.), Pd_2_(dba)_3_·CHCl_3_ (0.1 equiv.), and ^Me^CgPPh (0.4 equiv.) were dissolved in toluene under argon. The reaction mixture was stirred at room temperature for the reported time (see Table 3). Then, the reaction solution was loaded onto a SiO_2_ column wetted with hexane and eluted with an appropriate solvent (indicated below) to obtain the cross-coupling product. The monocrystals applicable for XRD were prepared by slow diffusion of *n*-heptane into the CH_2_Cl_2_ solution at 5 °C. The results are summarized in Table 3 (see Section 2).

#### 3.5.1. Synthesis of 2,5-bis-(4,4,5,5-Tetramethyl-3-oxide-1-oxyl-4,5-dihydro-1*H*-imidazol-2-yl)-thiophene (**1**) [31]

NN–AuPPh_3_ (0.035 g, 2.1 equiv.), 2,5-diiodothiophene (0.0080 g, 1.0 equiv.), Pd_2_(dba)_3_·CHCl_3_ (0.0050 g, 0.2 equiv.), and ^Me^CgPPh (0.0060 g, 0.8 equiv.) were stirred in toluene (1 mL) for 24 h at room temperature; eluent hexane/EtOAc (1:4). Yield 0.0088 g (92%), green crystals, mp 207–208 °C. 

#### 3.5.2. Synthesis of 2-(5-Fluorothiophen-2-yl)-4,4,5,5-tetramethyl-4,5-dihydro-1*H*-imidazol-3-oxide-1-oxyl (**2**)

NN–AuPPh_3_ (0.090 g, 1.0 equiv.), 2-fluoro-5-iodothiophene (0.033 g, 1.0 equiv.), Pd_2_(dba)_3_·CHCl_3_ (0.0150 g, 0.1 equiv.), and ^Me^CgPPh (0.0170 g, 0.4 equiv.) were stirred in toluene (2 mL) for 20 h at room temperature; eluent hexane/EtOAc (4:1). Yield 0.0340 g (91%), blue crystals, mp 98–100 °C. FTIR (KBr, cm^−1^) 450, 511, 540, 571, 619, 645, 707, 747, 786, 804, 872, 895, 961, 1010, 1026, 1139, 1199, 1277, 1328, 1371, 1402, 1444, 1526, 1565, 1602, 2875, 2936, 2988, 3134, 3444. Elemental analysis found (%) C 51.55, H 5.37, N 10.77; calculated for C_11_H_14_N_2_O_2_FS: C 51.35, H 5.48, N 10.89.

Comparative experiment: NN–AuPPh_3_ (0.030 g, 1.0 equiv.), 2-fluoro-5-iodothiophene (0.011 g, 1.0 equiv.), and Pd(PPh_3_)_4_ (0.011 g, 0.2 equiv.) were stirred in toluene (1 mL) for 20 h at room temperature; eluent hexane/EtOAc (4:1). Yield 0.0030 g (24%).

#### 3.5.3. Synthesis of 2-(4,6-Difluorobenzo[d]thiazole-2-yl)-4,4,5,5-tetramethyl-4,5-dihydro-1*H*-imidazol-3-oxide-1-oxyl (**3**)

NN–AuPPh_3_ (0.105 g, 1.0 equiv.), 4,6-difluoro-2-iodobenzo[*d*]thiazole (0.050 g, 1.0 equiv.), Pd_2_(dba)_3_·CHCl_3_ (0.0170 g, 0.1 equiv.), and ^Me^CgPPh (0.0200 g, 0.4 equiv.) were stirred in toluene (3 mL) for 24 h at room temperature; eluent hexane/EtOAc (2:1). Yield 0.0430 g (78%), bluish-green crystals, mp 195–196 °C. FTIR (KBr, cm^−1^) 437, 490, 540, 571, 608, 638, 684, 737, 783, 835, 852, 880, 911, 958, 995, 1077, 1112, 1142, 1179, 1218, 1255, 1287, 1311, 1329, 1375, 1429, 1451, 1469, 1525, 1575, 1598, 1620, 1653, 1685, 2110, 2540, 2645, 2949, 2993, 3017, 3074, 3092. Elemental analysis found (%) C 51.37, H 4.12, N 12.94; calculated for C_14_H_14_N_3_O_2_F_2_S: C 51.53, H 4.32, N 12.88.

#### 3.5.4. Synthesis of 2-(6-Fluorobenzo[d]thiazole-2-yl)-4,4,5,5-tetramethyl-4,5-dihydro-1*H*-imidazol-3-oxide-1-oxyl (**4**)

NN–AuPPh_3_ (0.110 g, 1.0 equiv.), 6-fluoro-2-iodobenzo[*d*]thiazole (0.050 g, 1.0 equiv.), Pd_2_(dba)_3_·CHCl_3_ (0.0180 g, 0.1 equiv.), and ^Me^CgPPh (0.0210 g, 0.4 equiv.) were stirred in toluene (3 mL) for 24 h at room temperature; eluent hexane/EtOAc (2:1). Yield 0.0380 g (70%), bluish-green crystals, mp 175–176 °C. FTIR (KBr, cm^−1^) 439, 493, 540, 555, 593, 609, 631, 675, 692, 738, 809, 829, 843, 874, 917, 960, 1009, 1045, 1083, 1113, 1138, 1176, 1201, 1218, 1247, 1270, 1318, 1374, 1424, 1452, 1470, 1528, 1563, 1604, 1652, 1681, 1740, 1867, 2876, 2938, 2990, 3080, 3444. Elemental analysis found (%) C 54.47, H 4.67, N 13.80; calculated for C_14_H_15_N_3_O_2_FS: C 54.53, H 4.90, N 13.63.

#### 3.5.5. Synthesis of 2-(1-Methylpyrazol-4-yl)-4,4,5,5-tetramethyl-4,5-dihydro-1*H*-imidazol-3-oxide-1-oxyl (**5**)

NN–AuPPh_3_ (0.030 g, 1.0 equiv.), 4-iodo-1-methylpyrazole (0.010 g, 1.0 equiv.), Pd_2_(dba)_3_·CHCl_3_ (0.0050 g, 0.1 equiv.), and ^Me^CgPPh (0.0060 g, 0.4 equiv.) were stirred in toluene (1 mL) for 18 h at room temperature; eluent EtOAc. Yield 0.0095 g (82%), blue crystals, mp 172–172.5 °C.

#### 3.5.6. Synthesis of 1,4-bis(4-(4,4,5,5-Tetramethyl-3-oxide-1-oxyl-4,5-dihydro-1*H*-imidazol-2-yl)pyrazol-1-yl)benzene (**6**) [32]

NN–AuPPh_3_ (0.032 g, 2.1 equiv.), 1,4-bis(4-iodopyrazol-1-yl)benzene (0.011 g, 1.0 equiv.), Pd_2_(dba)_3_·CHCl_3_ (0.0050 g, 0.2 equiv.), and ^Me^CgPPh (0.0060 g, 0.8 equiv.) were stirred in toluene (1 mL) for 20 h at room temperature; eluent hexane/EtOAc (1:2). Yield 0.0070 g (65%), blue crystals, mp 89–91 °C. Elemental analysis found (%) C 59.13, H 5.82, N 20.97; calculated for C_26_H_32_N_8_O_4_: C 59.99, H 6.20, N 21.52.

#### 3.5.7. Synthesis of 2-(1-Methyl-3-nitro-pyrazole-4-yl)-4,4,5,5-tetramethyl-4,5-dihydro-1*H*-imidazol-3-oxide-1-oxyl (**7**)

NN–AuPPh_3_ (0.090 g, 1.0 equiv.), 4-iodo-1-methyl-3-nitropyrazole (0.037 g, 1.0 equiv.), Pd_2_(dba)_3_·CHCl_3_ (0.0150 g, 0.1 equiv.), and ^Me^CgPPh (0.0170 g, 0.4 equiv.) were stirred in toluene (2 mL) for 48 h at room temperature; eluent EtOAc. Yield 0.0180 g (44%), dark emerald crystals, mp 170–172 °C. FTIR (KBr, cm^−1^) 424, 441, 467, 506, 539, 604, 631, 647, 676, 695, 722, 753, 764, 805, 849, 873, 978, 997, 1015, 1029, 1078, 1118, 1139, 1175, 1224, 1298, 1334, 1361, 1401, 1438, 1453, 1481, 1496, 1544, 1589, 1613, 1674, 1699, 2871, 2946, 2984, 2999, 3016, 3035, 3054, 3075, 3104, 3134, 3467, 3500. Elemental analysis found (%) C 46.43, H 5.60, N 24.72; calculated for C_11_H_16_N_5_O_4_: C 46.80, H 5.71, N 24.81.

#### 3.5.8. Synthesis of 2-(1-Difluoromethylpyrazol-5-yl)-4,4,5,5-tetramethyl-4,5-dihydro-1*H*-imidazol-3-oxide-1-oxyl (**8**)

NN–AuPPh_3_ (0.090 g, 1.0 equiv.), 1-(difluoromethyl)-5-iodopyrazole (0.036 g, 1.0 equiv.), Pd_2_(dba)_3_·CHCl_3_ (0.0150 g, 0.1 equiv.), and ^Me^CgPPh (0.0170 g, 0.4 equiv.) were stirred in toluene (2 mL) for 24 h at room temperature; eluent hexane/EtOAc (1:1). Yield 0.0320 g (80%), blue crystals, mp 182–183 °C. FTIR (KBr, cm^−1^) 414, 474, 493, 542, 556, 596, 619, 643, 671, 745, 794, 829, 857, 918, 930, 968, 1003, 1041, 1055, 1072, 1131, 1145, 1167, 1195, 1222, 1263, 1288, 1303, 1321, 1349, 1374, 1395, 1415, 1437, 1458, 1487, 1579, 1651, 2872, 2941, 2992, 3053, 3113, 3130, 3151, 3452. Elemental analysis found (%) C 48.23, H 5.49, N 20.61; calculated for C_11_H_15_N_4_O_2_F_2_: C 48.35, H 5.53, N 20.50.

#### 3.5.9. Synthesis of 2-(1-Isopropylpyrazol-3-yl)-4,4,5,5-tetramethyl-4,5-dihydro-1*H*-imidazol-3-oxide-1-oxyl (**9**)

NN–AuPPh_3_ (0.090 g, 1.0 equiv.), 3-iodo-1-isopropylpyrazole (0.035 g, 1.0 equiv.), Pd_2_(dba)_3_·CHCl_3_ (0.0150 g, 0.1 equiv.), and ^Me^CgPPh (0.0170 g, 0.4 equiv.) were stirred in toluene (2 mL) for 48 h at room temperature; eluent hexane/EtOAc (1:2). Yield 0.0180 g (47%), blue crystals, mp 198–199 °C. FTIR (KBr, cm^−1^) 411, 462, 501, 541, 602, 658, 700, 722, 752, 791, 813, 869, 884, 935, 967, 984, 1007, 1057, 1087, 1137, 1167, 1186, 1214, 1254, 1339, 1373, 1411, 1451, 1488, 1501, 1562, 1678, 1778, 2815, 2872, 2931, 2973, 3102, 3157, 3461. Elemental analysis found (%) C 58.61, H 7.83, N 21.02; calculated for C_13_H_21_N_4_O_2_: C 58.85, H 7.98, N 21.12.

#### 3.5.10. Synthesis of 2-(1-Methylpyrazol-3-yl)-4,4,5,5-tetramethyl-4,5-dihydro-1*H*-imidazol-3-oxide-1-oxyl (**10**)

NN–AuPPh_3_ (0.030 g, 1.0 equiv.), 3-iodo-1-methylpyrazole (0.010 g, 1.0 equiv.), Pd_2_(dba)_3_·CHCl_3_ (0.0050 g, 0.1 equiv.), and ^Me^CgPPh (0.0060 g, 0.4 equiv.) were stirred in toluene (1 mL) for 72 h at room temperature; eluent hexane/acetone (1:2). Yield 0.0030 g (26%), blue crystals, mp 179.5–180.5 °C.

#### 3.5.11. Synthesis of 2-(5-Ethylisoxazole-4-yl)-4,4,5,5-tetramethyl-4,5-dihydro-1*H*-imidazol-3-oxide-1-oxyl (**11**)

NN–AuPPh_3_ (0.090 g, 1.0 equiv.), 5-ethyl-4-iodoisoxazole (0.033 g, 1.0 equiv.), Pd_2_(dba)_3_·CHCl_3_ (0.0150 g, 0.1 equiv.), and ^Me^CgPPh (0.0170 g, 0.4 equiv.) were stirred in toluene (2 mL) for 24 h at room temperature; eluent CH_2_Cl_2_/EtOAc (3:1). Yield 0.0185 g (50%), violet crystals, mp 160–162 °C. FTIR (KBr, cm^−1^) 439, 502, 540, 567, 585, 622, 692, 718, 748, 791, 813, 853, 894, 923, 976, 997, 1027, 1045, 1070, 1101, 1137, 1178, 1216, 1263, 1344, 1368, 1406, 1436, 1459, 1480, 1543, 1593, 1626, 1684, 1703, 1721, 1753, 1812, 1897, 1970, 2144, 2173, 2339, 2363, 2683, 2856, 2874, 2927, 2966, 3057, 3449. Elemental analysis found (%) C 56.37, H 6.71, N 16.08; calculated for C_12_H_18_N_3_O_3_: C 57.13, H 7.19, N 16.66.

#### 3.5.12. Synthesis of 2-(3,5-Dimethylisoxazole-4-yl)-4,4,5,5-tetramethyl-4,5-dihydro-1*H*-imidazol-3-oxide-1-oxyl (**12**) [33]

NN–AuPPh_3_ (0.030 g, 1.0 equiv.), 4-iodo-3,5-dimethylisoxazole (0.011 g, 1.0 equiv.), Pd_2_(dba)_3_·CHCl_3_ (0.0050 g, 0.1 equiv.), and ^Me^CgPPh (0.0060 g, 0.4 equiv.) were stirred in toluene (1 mL) for 72 h at room temperature; eluent CH_2_Cl_2_/EtOAc (3:1). Yield 0.0050 g (42%), violet crystals, mp 172–174 °C.

#### 3.5.13. Synthesis of 5-(4,4,5,5-Tetramethyl-3-oxide-1-oxyl-4,5-dihydro-1*H*-imidazol-2-yl)-4-chloro-1-methyl-1-phenyl-2-pyrimidineamine (**13**)

NN–AuPPh_3_ (0.090 g, 1.0 equiv.), 4-chloro-5-iodo-1-methyl-1-phenyl-2-pyrimidineamine (0.055 g, 1.0 equiv.), Pd_2_(dba)_3_·CHCl_3_ (0.0150 g, 0.1 equiv.), and ^Me^CgPPh (0.0170 g, 0.4 equiv.) were stirred in toluene (1 mL) for 48 h at room temperature; eluent CH_2_Cl_2_/EtOAc (3:1). Yield 0.0235 g (69%), violet crystals, mp 162–164 °C. FTIR (KBr, cm^−1^) 431, 452, 497, 513, 539, 590, 623, 644, 702, 749, 781, 803, 865, 925, 949, 976, 1027, 1073, 1090, 1130, 1164, 1207, 1261, 1343, 1365, 1396, 1415, 1450, 1502, 1534, 1590, 1606, 2865, 2937, 2964, 2989, 3054, 3456. Elemental analysis found (%) C 57.66, H 4.74, N 18.08; calculated for C_18_H_21_ClN_5_O_2_: C 57.68, H 5.65, N 18.68.

#### 3.5.14. Synthesis of 2-(Pyridin-4-yl)-4,4,5,5-tetramethyl-4,5-dihydro-1*H*-imidazol-3-oxide-1-oxyl (**14**) [14]

NN–AuPPh_3_ (0.030 g, 1.0 equiv.), 4-iodopyridine (0.010 g, 1.0 equiv.), Pd_2_(dba)_3_·CHCl_3_ (0.0050 g, 0.1 equiv.), and ^Me^CgPPh (0.0055 g, 0.4 equiv.) were stirred in toluene (1 mL) for 16 h at room temperature; eluent hexane/EtOAc (1:5). Yield 0.0105 g (92%), violet crystals, mp 127–129 °C.

### 3.6. X-ray Crystallography Details

The sets of data for single crystals of **2**–**4**, **7**–**9**, **11**, **13** were collected on a Bruker automated diffractometers Kappa Apex II CCD (MoKα), Smart Apex (MoKα), and Apex Duo (Cu Kα) at room temperature using φ-ω scans. Absorption correction was applied by means of Bruker SADABS (version 2.10) [34]. The structure was solved by direct methods and refined by the full-matrix least-squares method in an anisotropic approximation for all nonhydrogen atoms. Positions of the H atoms were calculated geometrically and included in the refinement in a riding model. All calculations for structure solution and refinement were performed in SHELXL-2018/3 [35,36]. Crystallographic characteristics of nitronyl nitroxides and details of the SC XRD experiments are listed in Table S1, selected stereochemical characteristics of nitronyl nitroxides are presented in Table S2, and short contacts are presented in Table S3. Crystallographic data were deposited at the Cambridge Crystallographic Data Center (CCDC Nos. 2301486–2301492 and 2300480) and can be obtained free of charge via www.ccdc.cam.ac.uk/data_request/cif (accessed on 15 November 2023).

## 4. Conclusions

In this work, we devised a new methodology for the cross-coupling of heteroaryl iodides with NN–AuPPh_3_ by means of a catalytic system consisting of Pd_2_(dba)_3_·CHCl_3_ and ^Me^CgPPh. The usefulness of the protocol is that it allows for the cross-coupling reactions to be conducted at room temperature with high efficiency. The present protocol is well compatible with various heteroaryl iodides, and, in combination with the previously developed reagents NN–AuL (L = XPhos, ^Me^CgPPh, or TTMPP) for cross-coupling with aryl bromides, opens new horizons in directed synthesis of functionalized nitronyl nitroxides. Application of both new cross-coupling methodologies to the preparation of hyper-high-spin molecules is underway in our laboratory.

## Figures and Tables

**Table 1:** Effects of phosphine ligands on the cross-coupling of *p*-BrC_6_H_4_OMe with NN–AuPPh_3_.

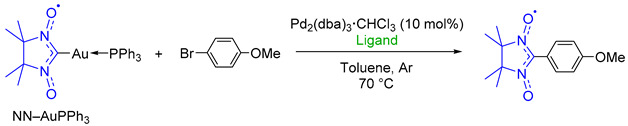			
**Entry**	**Ligand (40 mol%)**	**Reaction Time**	**Yield (%) ^a^**
1	none	6 h	traces
2	^Me^CgPPh	40 min	72
3	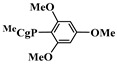	6 h	65
4	dppf (20 mol%)	3 h	47
5	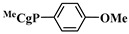	2 h	40
6	XPhos	4 h	35
7	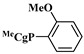	4 h	33
8	^t^Bu_3_P	7 h	30
9	^Me^CgP–CH_2_–OH	3 h	25
10	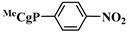	5 h	18
11	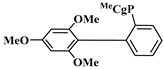	5 h	17
12	SPhos	7 h	traces
13	TTMPP	6 h	traces
14	rac–BINAP (20 mol%)	10 h	traces
15	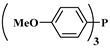	5 h	traces
16	(4-Tol)_3_P	5 h	traces
17	dppf (10 mol%)	3 h	traces
18	(C_6_F_5_)PPh_2_	5 h	traces
19	(C_6_F_5_)_2_PPh	5 h	traces
20	(C_6_F_5_)_3_P	5 h	traces
21	Ph_3_P	5 h	traces

^a^ Here and below, all product yields are calculated for compounds isolated in pure form.

**Table 2:** Effects of phosphine ligands on the cross-coupling of *p*-IC_6_H_4_OMe with NN–AuPPh_3_.

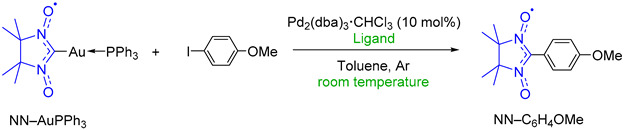			
**Entry**	**Ligand (40 mol%)**	**Reaction Time**	**Yield (%)**
1	none	24 h	traces
2	^Me^CgPPh	18 h	90
3	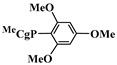	24 h	82
4	dppf (20 mol%)	24 h	71
5	XPhos	24 h	84
6	(4-Tol)_3_P	24 h	68

**Table 3:** Cross-coupling reactions of heteroaryl iodides with NN–AuPPh_3_.

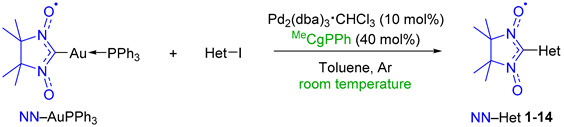				
**Entry**	**Het**–**I**	**Nitronyl Nitroxide 1–14**	**Reaction Time**	**Yield (%)**
1	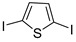	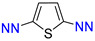	24 h	92
2	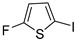	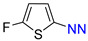	20 h	91
3	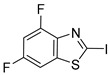	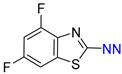	18 h	78
4	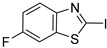	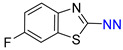	18 h	70
5	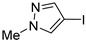	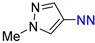	18 h	82
6	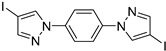	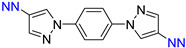	20 h	65
7	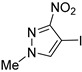	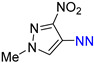	48 h	44
8	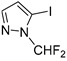	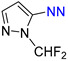	24 h	80
9	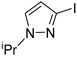	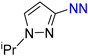	48 h	47
10	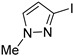	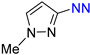	72 h	26
11	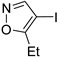	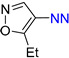	24 h	50 45 (20 mol% ^Me^CgPPh) 25 (80 mol% ^Me^CgPPh)
12	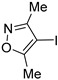	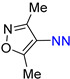	72 h	42
13	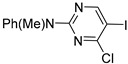	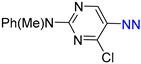	48 h	69
14	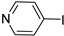	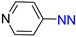	16 h	92

## Data Availability

The data that support the findings of this study are available upon reasonable request from the authors.

## Supplementary Materials

The following supporting information can be downloaded at: https://www.mdpi.com/article/10.3390/molecules28227661/s1, Tables S1–S3: Crystallographic characteristics, bond lengths, and short contacts of compounds **2**–**4**, **7**–**9**, **11**, **13**; Figures S1–S8: Structures of compounds **2**–**4**, **7**–**9**, **11**, **13**; Figure S9: EPR spectra of compounds **2**–**4**, **7**–**9**, **11**, **13**; Table S4: Hyperfine interaction constants; Figures S10–S17: FT-IR spectra of compounds **2**–**4**, **7**–**9**, **11**, **13**.
