# Supplementary material for: Catalytic System for Cross-Coupling of Heteroaryl Iodides with a Nitronyl Nitroxide Gold Derivative at Room Temperature
Source: Molecules. 2023 Nov 19;28(22):7661. doi: 10.3390/molecules28227661 (PMC10675334; doi:10.3390/molecules28227661)
Supplement: Supplementary file 1 [file molecules-28-07661-s001.zip › molecules-2709223-supplementary.pdf]

Supporting Information

Article

Catalytic System for Cross-Coupling of Heteroaryl Iodides with a Nitronyl Nitroxide Gold Derivative at Room Temperature

Igor Zayakin [1], Galina Romanenko [2], Irina Bagryanskaya [3], Bogdan Ugrak [1], Matvey Fedin [2,*] and Evgeny Tretyakov [1,*]

[1] N. D. Zelinsky Institute of Organic Chemistry, Leninsky Ave. 47, Moscow 119991, Russia; zavnsc3@gmail.com (I.Z.); ugrak@ioc.ac.ru (B.U.)

[2] International Tomography Center, Institutskaya Str. 3a, Novosibirsk 630090, Russia; romanenko@tomo.nsc.ru

[3] N. N. Vorozhtsov Institute of Organic Chemistry, 9 Ac. Lavrentiev Avenue, Novosibirsk 630090, Russia; bagryan@nioch.nsc.ru

[*] Correspondence: mfedin@tomo.nsc.ru (M.F.); tretyakov@ioc.ac.ru (E.T.)

Table of Contents

Section S1. X-ray Crystallography....... 2

Section S2. EPR Spectra....... 12

Section S3. FT-IR Spectra....... 14

Section S1. X-ray Crystallography

Table S1. Crystallographic characteristics of nitronyl nitroxides and details of XRD experiments.

**Nitronyl nitroxide**	**2**	**3**	**4**	**7**
Formula	$C_{11}H_{14}FN_2O_2S$	$C_{14}H_{14}F_2N_3O_2S$	$C_{14}H_{15}FN_3O_2S$	$C_{11}H_{16}N_5O_4$
FW	257.30	326.34	308.35	282.29
T, K	296	296	296	296
Space group, *Z*	*P*-1, 4	*Pbcn*, 8	*C*2/*c*, 8	$P2_1/c$, 4
a,<br>*b*,<br>*c*, Å	10.2925(17)<br>11.1336(17)<br>13.117(2)	19.5360(12)<br>9.9325(7)<br>14.8554(11)	19.5596(12)<br>11.4392(12)<br>13.9099(9)	7.0474(5)<br>19.8419(12)<br>10.0584(7)
α,<br>β,<br>γ, °	73.496(11)<br>80.626(12)<br>62.970(10)	90<br>90<br>90	90<br>112.147(3)<br>90	90<br>105.829(4)<br>90
V, Å^3	1282.7(4)	2882.6(3)	2882.7(4)	1353.17(16)
d_{calc}, g·cm^3	1.332	1.504	1.421	1.386
θ_{max}, °	28.536	28.777	26.018	28.244
I_{hkl} meas / unique<br>R_{int}	16276 / 6353<br>0.0436	15111 / 3678<br>0.0405	20454 / 2820<br>0.0500	12525 / 3141<br>0.0658
$I_{hk\ l}(I>2\sigma_I)$ / Ns	2595 / 354	2132 / 207	2031 / 193	1789 / 246
GooF	0.917	1.007	1.063	0.889
R_1 / wR_2 ($I>2\sigma_I$)	0.0675 / 0.1920	0.0449 / 0.1018	0.0643 / 0.1577	0.0401 /0.0918
CCDC	2301490	2301492	2300480	2301491

Table S1. Crystallographic characteristics of nitronyl nitroxides and details of XRD experiments (*continuation*).

**Nitronyl nitroxide**	**8**	**9**	**11**	**13**
Formula	$C_{11}H_{15}F_2N_4O_4$	$C_{13}H_{21}N_4O_2$	$C_{12}H_{18}N_3O_3$	$C_{18}H_{21}ClN_5O_2$
FW	273.27	265.34	252.29	374.85
T, K	296	296	296	296
Space group, *Z*	$P2_1/c$, 4	*Pnma*, 4	$P2_1/n$, 4	*P*-1, 2
a,<br>*b*,<br>*c*, Å	9.9782(9)<br>10.2314(9)<br>13.1164(13)	12.401(7)<br>9.379(5)<br>12.577(7)	11.0099(9)<br>7.2116(6)<br>16.7968(15)	7.6650(10)<br>11.5910(10)<br>12.065(2)
α,<br>β,<br>γ, °	90<br>110.670(7)<br>90	90<br>90<br>90	90<br>93.143(5)<br>90	114.711(7)<br>96.458(8)<br>100.801(8)
V, Å^3	1252.9(2)	1462.9(14)	1331.6(2)	934.4(2)
d_{calc}, g·cm^3	1.449	1.205	1.258	1.332
θ_{max}, °	28.446	28.633	28.335	28.804
I_{hkl} meas / unique<br>R_{int}	11864 / 3135<br>0.0495	8212 / 1621<br>0.0940	12169 / 3321 /<br>0.0277	4723 / 4723<br>0
$I_{hk\ l}(I>2\sigma_I)$ / Ns	1554 / 233	595 / 106	1851 / 236	1938 / 247
GooF	1.040	0.786	1.013	1.008
R_1 / wR_2 ($I>2\sigma_I$)	0.0391 / 0.0678	0.0638 / 0.1607	0.0456 / 0.0959	0.1053 / 0.3016
CCDC	2301488	2301486	2301489	2301487

Table S2. Selected stereochemical characteristics of nitronyl nitroxides (Å, deg).

Nitroxide		N–O	∀(N2-C11-C12-C(S)13)
**2**	A[a]	1.277(5), 1.280(4)	–2.0(4)
	B[a]	1.281(4), 1.284(5)	1.9(5)
**3**		1.273(2), 1.273(2)	1.4(3)
**4**		1.275(4), 1.250(4)	11.0(4)
**7**		1.282(2). 1.278(1)	32.4(3)
**8**		1.273(1), 1.283(2)	35.1(3)
**9**		1.276(5), 1.274(5)	0.0
**11**		1.280(2), 1.278(2)	–40.6(2)
**13**		1.284(5), 1.264(5)	61.1(7)

[a] Crystallographically independent molecules.

Table S3. Short contacts in the structures of nitronyl nitroxides (Å).

Radical	Intermolecular						Intramolecular
	O_{NO}…O_{NO}	O_{NO}…N	F…N	O_{NO}…N	O_{NO}…O_{NO2}	O_{NO}…$_{H}$C	
**2**	4.119(4)	3.962(4), 3.986(3)	3.562(2)			3.346(6)	
**3**	3.572(2), 3.873(2)	3.513(2)	3.467(2)				
**4**	3.905(6), 3.917(6)						
**7**	3.345(2)				3.315(2)		
**8**	3.371(2), 3.794(2)			3.365(2)			O_{NO}…F 3.042(1)
**9**	5.194(1)			4.788(1)		3.268(1), 3.154(1)	
**11**	4.016(2), 4.188(2), 4.410(2)						
**13**	5.066(8)			3.374(6)	3.069(3) Cl…Cl		

In all radicals, the structure of the nitronyl nitroxide moiety is almost identical. The N–O bond lengths of the paramagnetic moiety are in the range 1.273(2)–1.284(5) Å. Nitronyl nitroxides differ in the values of torsion angles between the planes of the paramagnetic ONCNO moiety and the aromatic heterocycle, which is due to the steric influence of vicinal substituents (Figures S1–S8). Thus, in nitronyl nitroxides **2**, **3**, and **9** without substituents in the aromatic cycle, these angles do not exceed 2º (Table S2), while in the rest radicals their values are more than 32º.

The structural features of heterocyclic substituents in nitronyl nitroxides **2**, **3**, **7**, **8**, **9**, **11** predetermine the diversity of intra- and intermolecular contacts in their structures (Table S3, Figures S1–S8). For example, in the crystal structures of paramagnets **2** and **3**, there are short F…N contacts (3.562 Å in **2** and 3.467(2) Å in **3**), and in the structures of radicals **2** and **9**, the molecules are linked in a chain via hydrogen bonds C–H…O_{NO}.

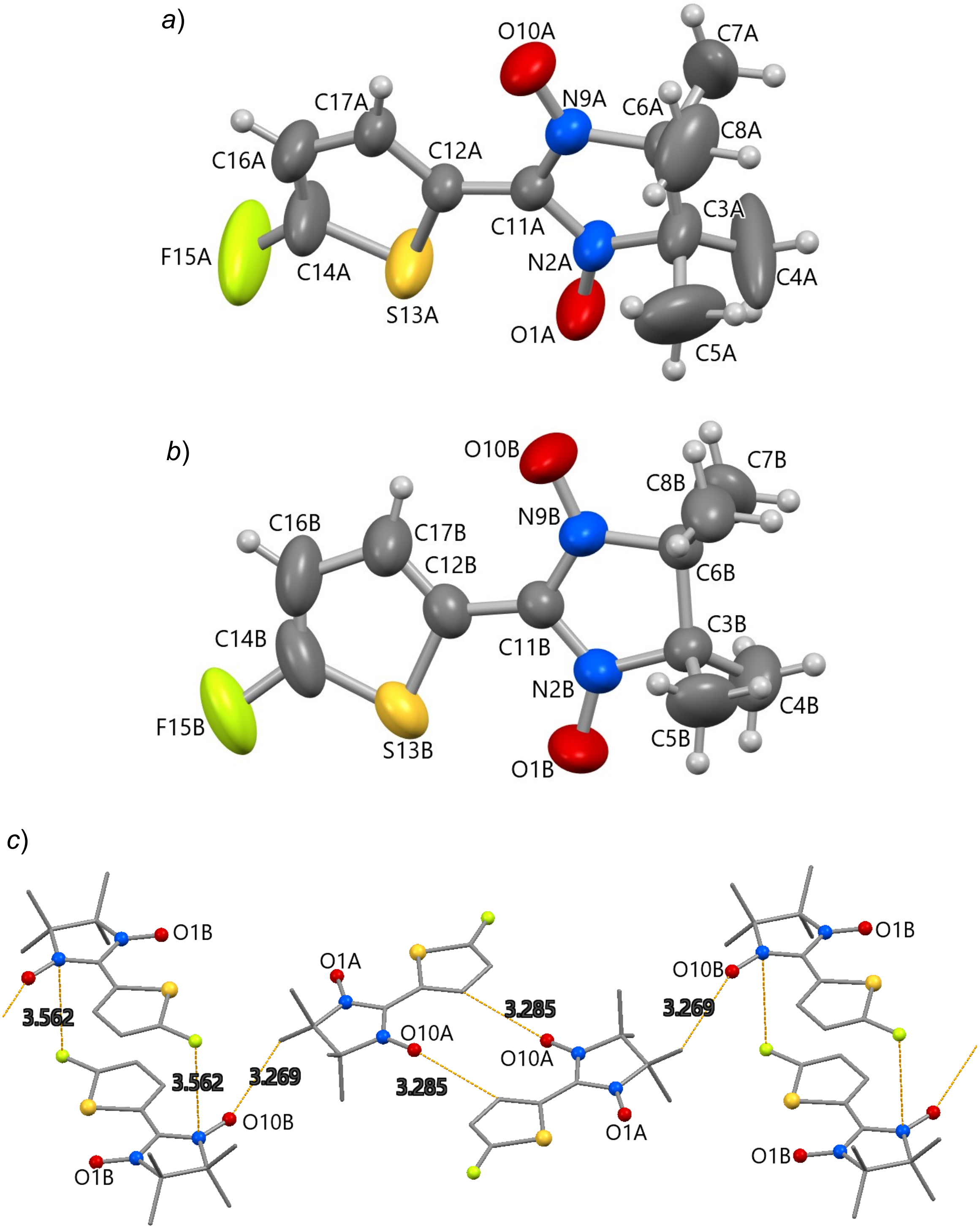

Figure S1. Structures of crystallographically independent molecules **2** (*a* and *b*) and short intermolecular contacts betweem molecules (*c*).

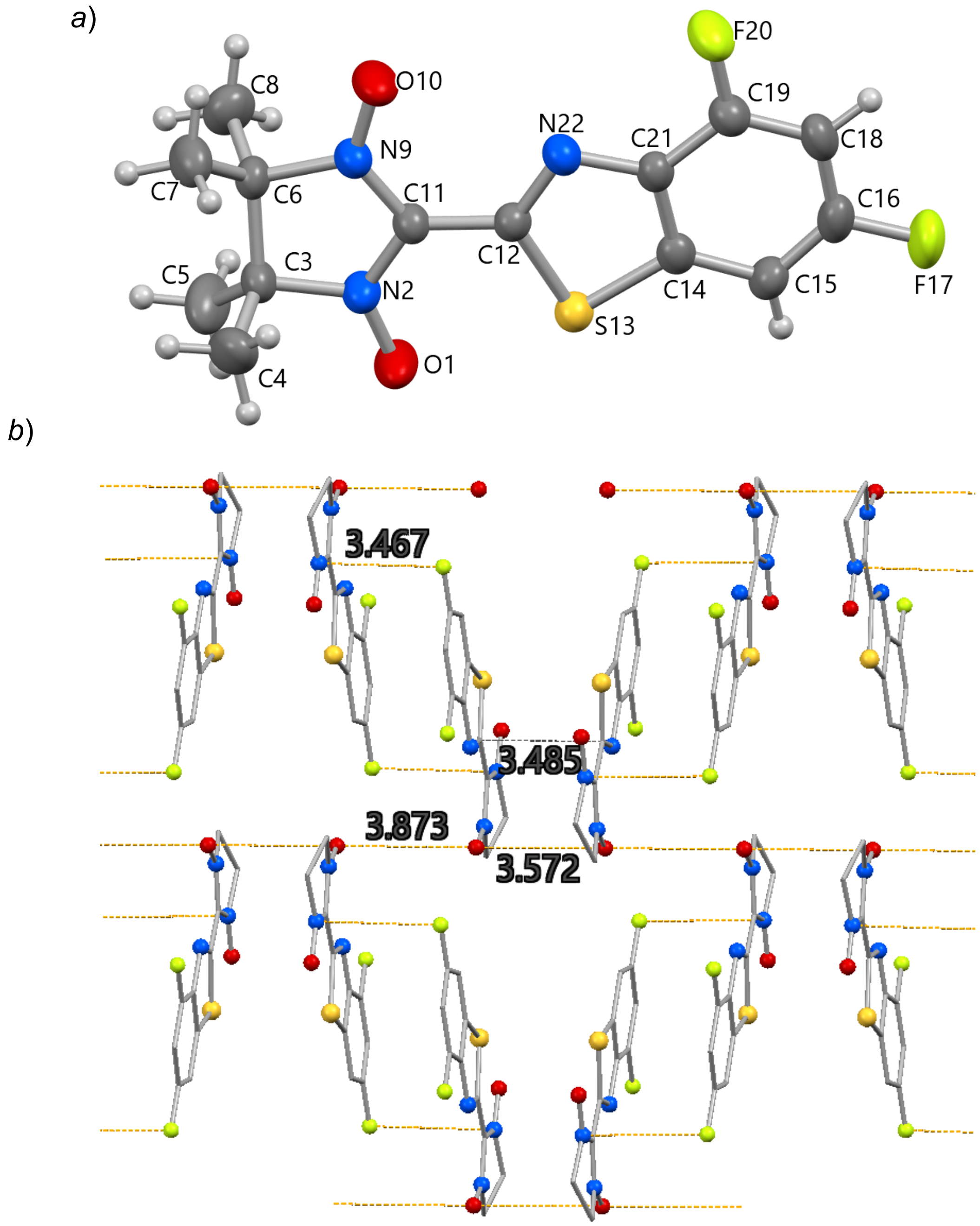

Figure S2. Structure of molecule **3** (*a*) and short intermolecular contacts in crystal structure (*b*).

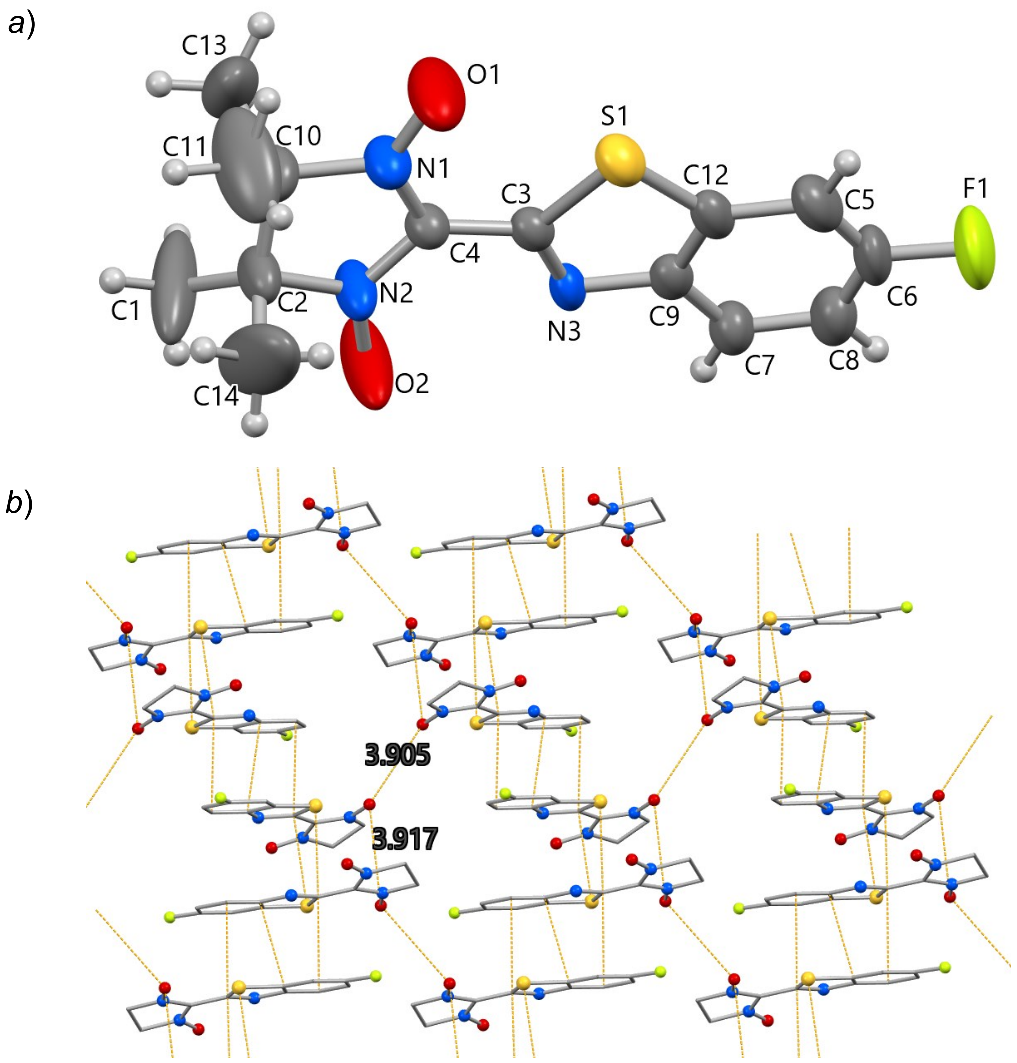

Figure S3. Structure of molecule **4** (*a*) and short intermolecular contacts in crystal structure (*b*).

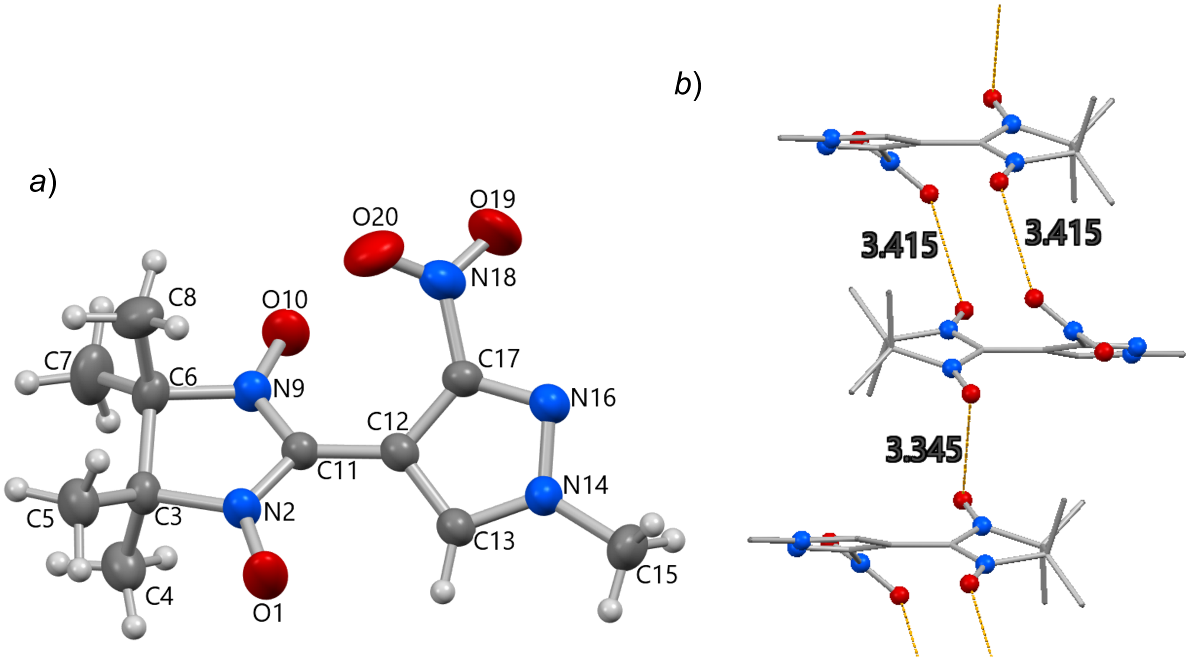

Figure S4. Structure of molecule **7** (*a*) and short intermolecular contacts in crystal structure (*b*).

A feature of crystal packing of nitronyl nitroxide **8** is the presence of a short intramolecular contact F…O_{NO} (3.042 Å) and relatively short O_{NO}…O_{NO} distances (3.371 and 3.794 Å), alternating in the chain of molecules (Figure S5, b).

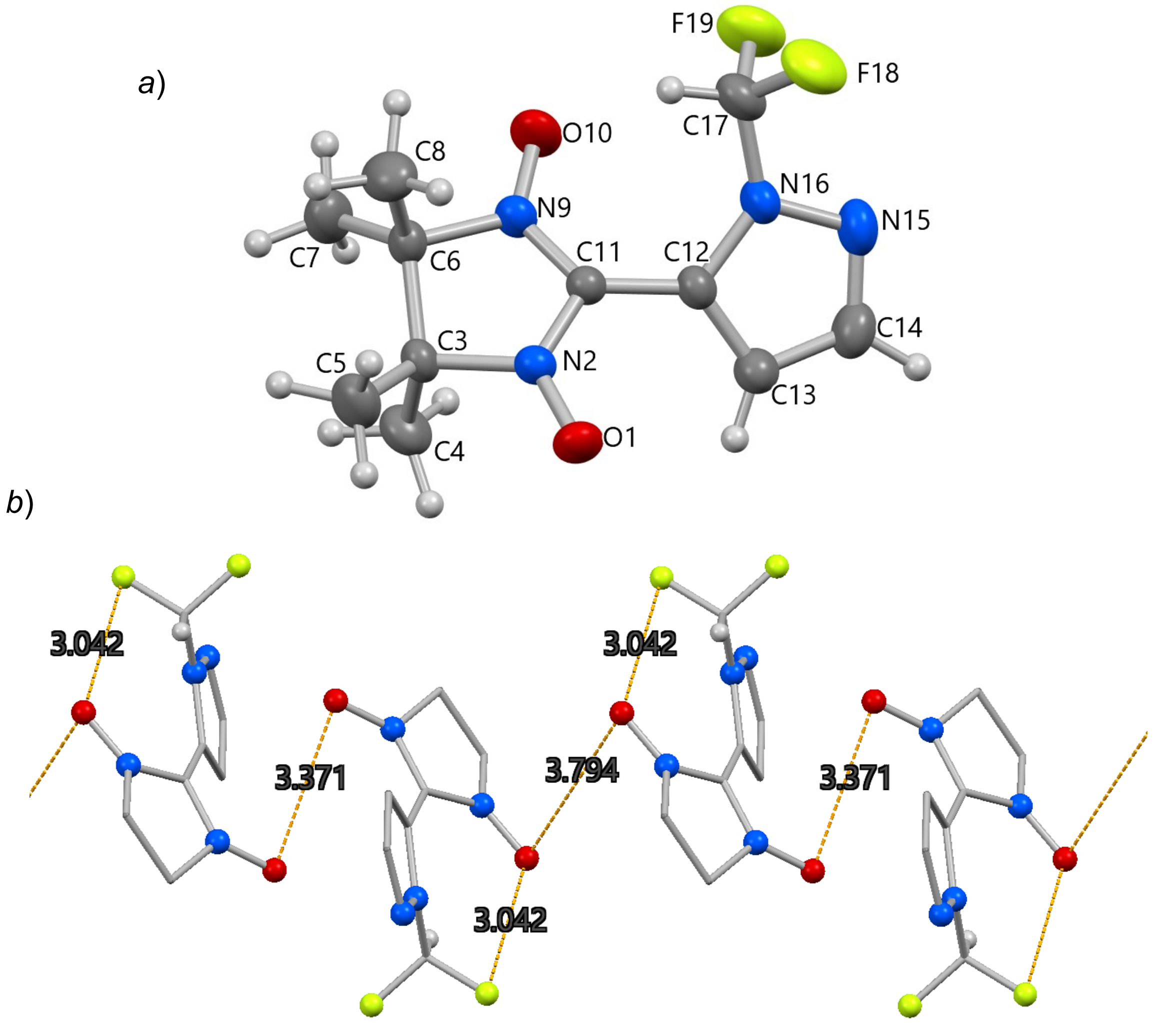

Figure S5. Structure of molecule **8** (*a*) and short intermolecular contacts in crystal structure (*b*).

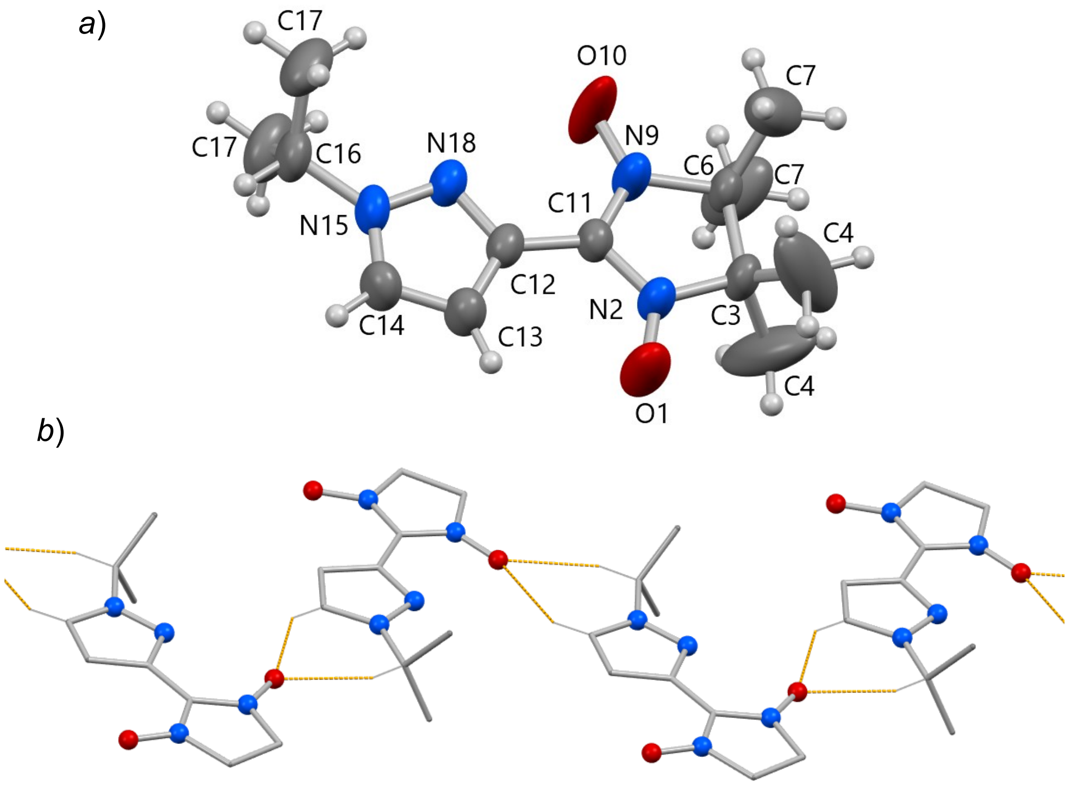

Figure S6. Structure of molecule **9** (*a*) and short intermolecular contacts in crystal structure (*b*).

In nitroxide **11**, the shortest contacts between paramagnetic centers exceed 4 Å (Figure S7), while in **7** the specific stacking of molecules makes it possible to distinguish dimers inside them with O…O_{NO} distances of 3.345(2) Å (Figure S4).

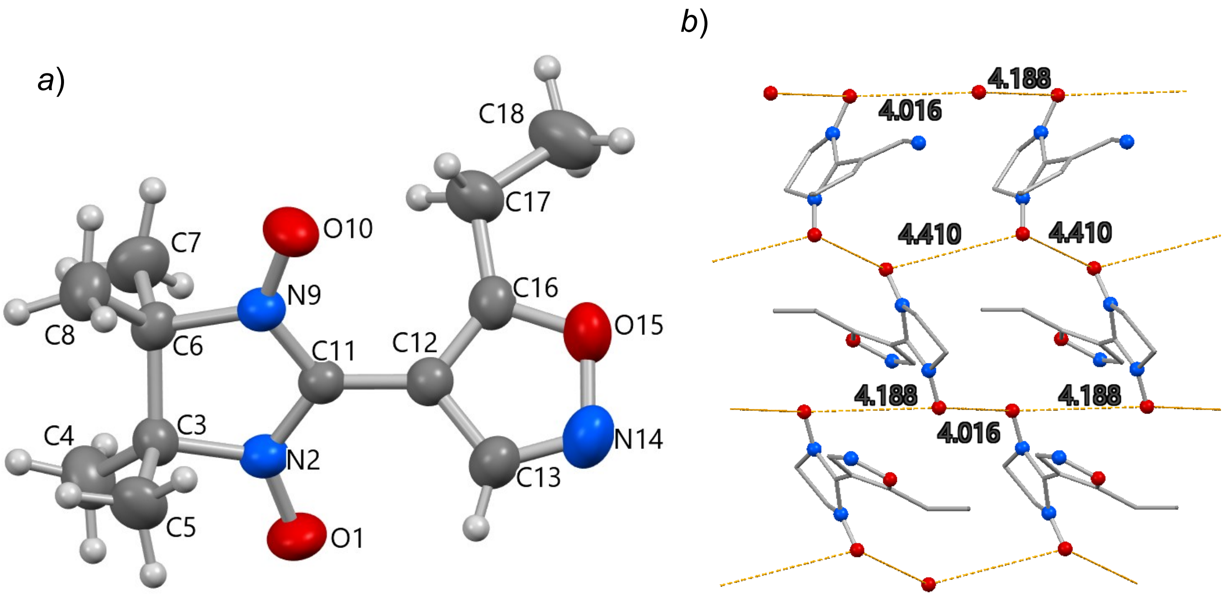

Figure S7. Structure of molecule **11** (*a*) and short intermolecular contacts in crystal structure (*b*).

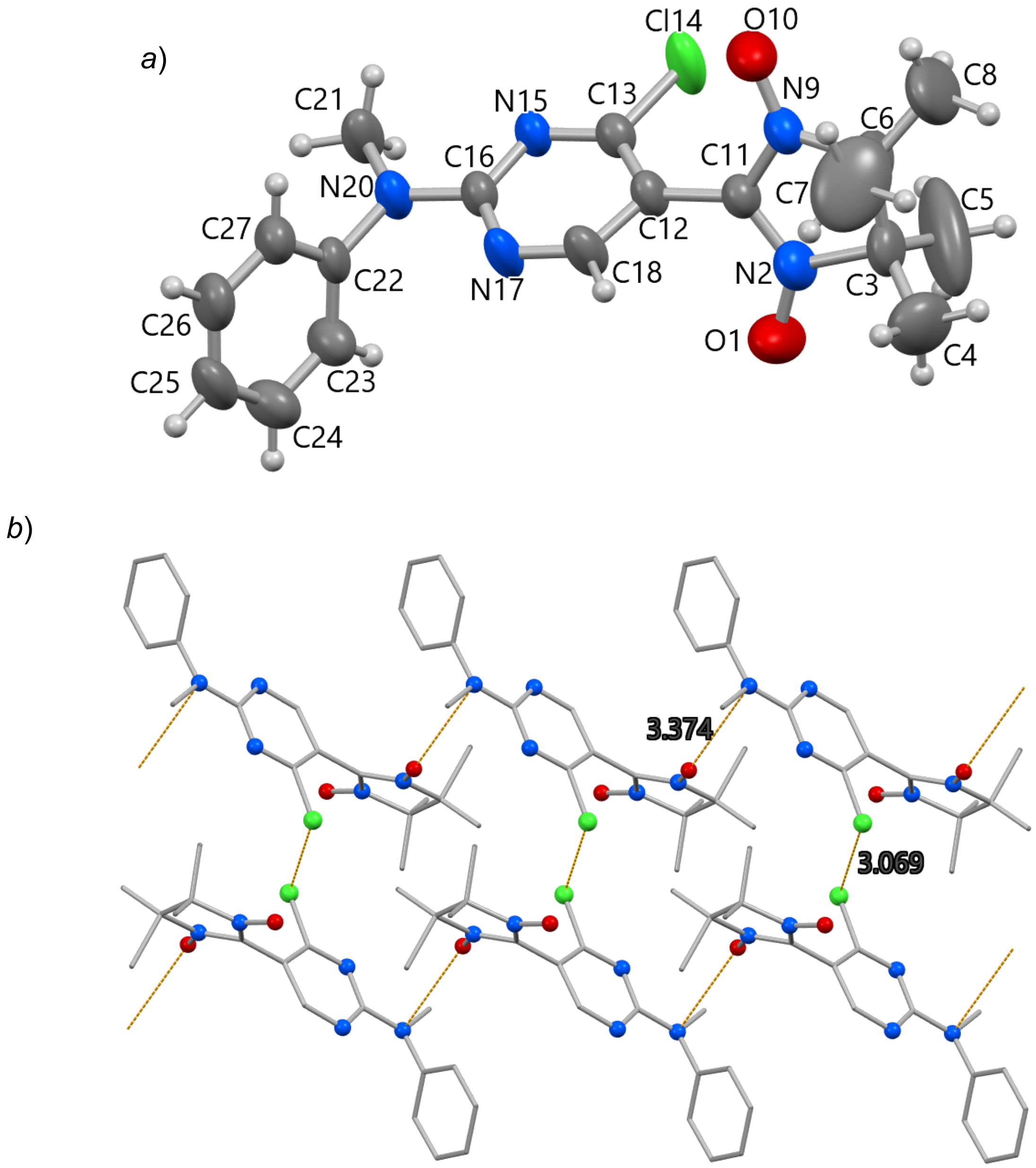

Figure S8. Structure of molecule **13** (*a*) and short intermolecular contacts in crystal structure (*b*).

Section S2. EPR Spectra

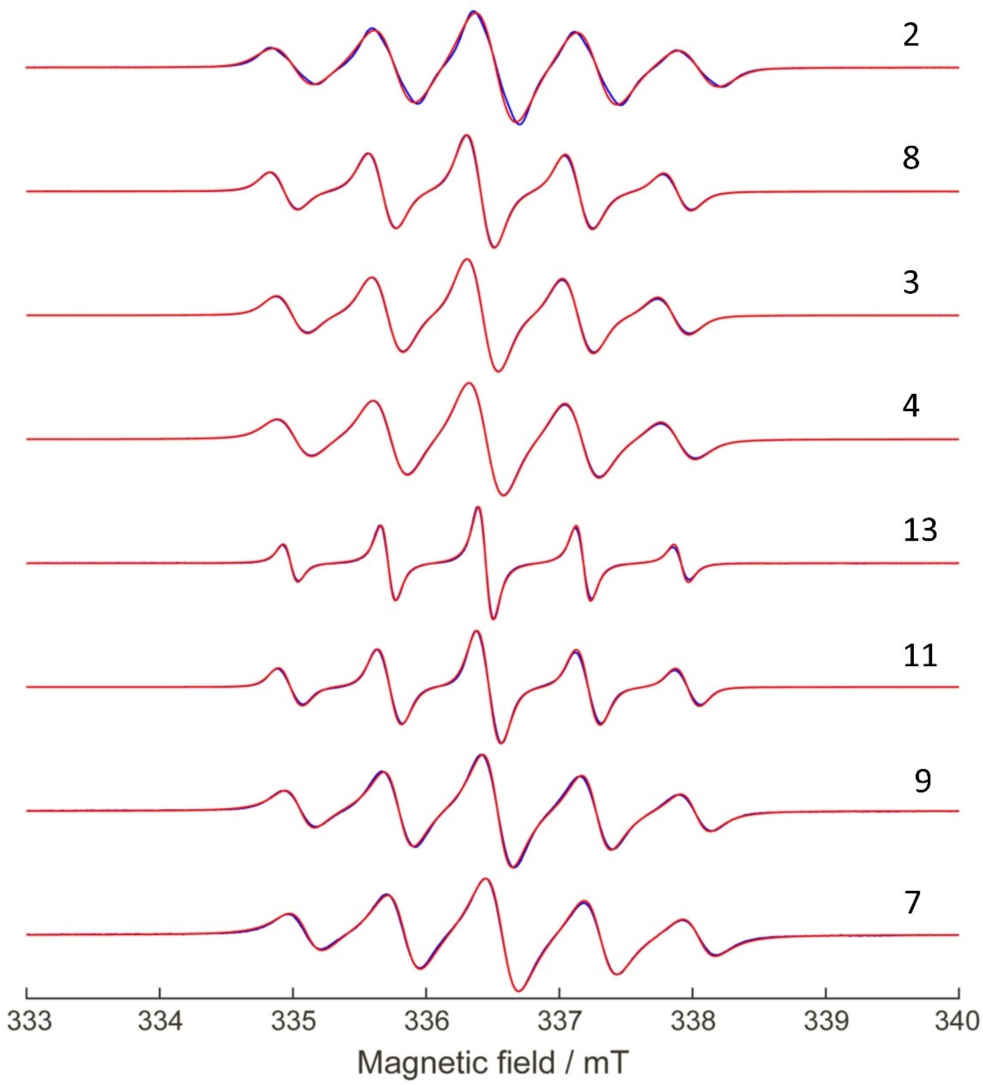

Figure S9. Continuous wave EPR spectra of nitronyl nitroxides (indicated in the plot) obtained in toluene solution at 290 K. Experimental traces are shown in blue, corresponding simulations – in red (see text for parameters).

All obtained EPR spectra were simulated using a model of a radical (S=1/2) with two equivalent ^{14}N hyperfine interaction constants and isotropic g-factor. Different linewidths were accounted by corresponding Gaussian and Lorentzian broadenings. Table S4 shows the values of g-factors and HFI constants used in simulations shown in Figure S9.

Table S4. Parameters used in simulations shown in Figure S9: ^{14}N isotropic hyperfine interaction constants (A) and corresponding g-factors.

Nitronyl nitroxide	**A / mT**	**g-factor**
**2**	0.761	2.007
**3**	0.717	2.007
**4**	0.720	2.007
**7**	0.738	2.007
**8**	0.740	2.007
**9**	0.744	2.007
**11**	0.747	2.007
**13**	0.735	2.007

As follows from Table S4, the obtained g-factors are nearly identical (within an accuracy of ~0.0002), and hyperfine interaction constants are also rather similar, being typical of nitronyl nitroxides.

Section S3. FT-IR Spectra

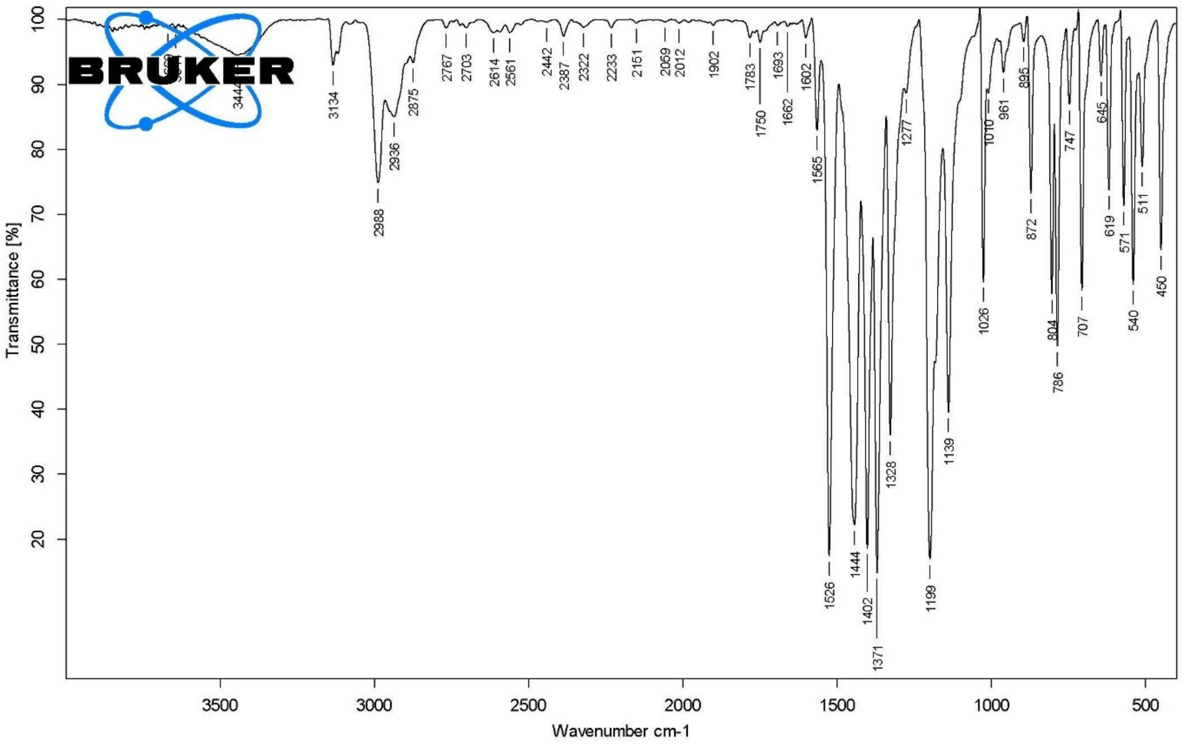

Figure S10. FT-IR spectrum of **2** in KBr.

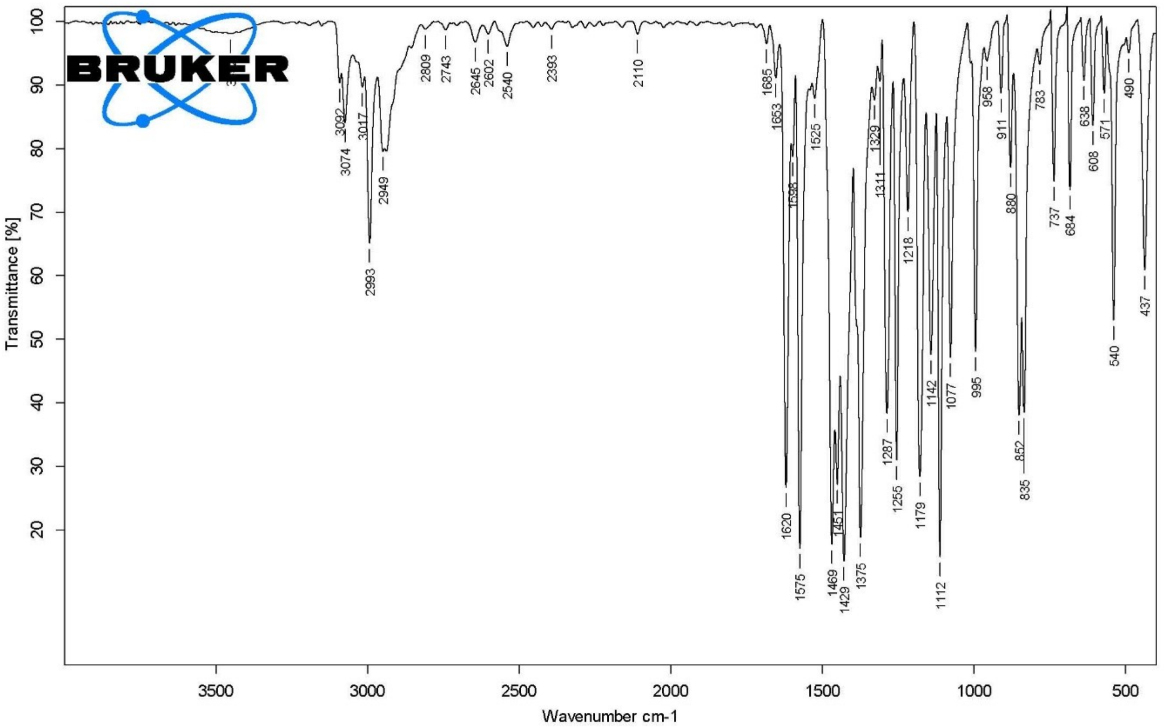

Figure S11. FT-IR spectrum of **3** in KBr.

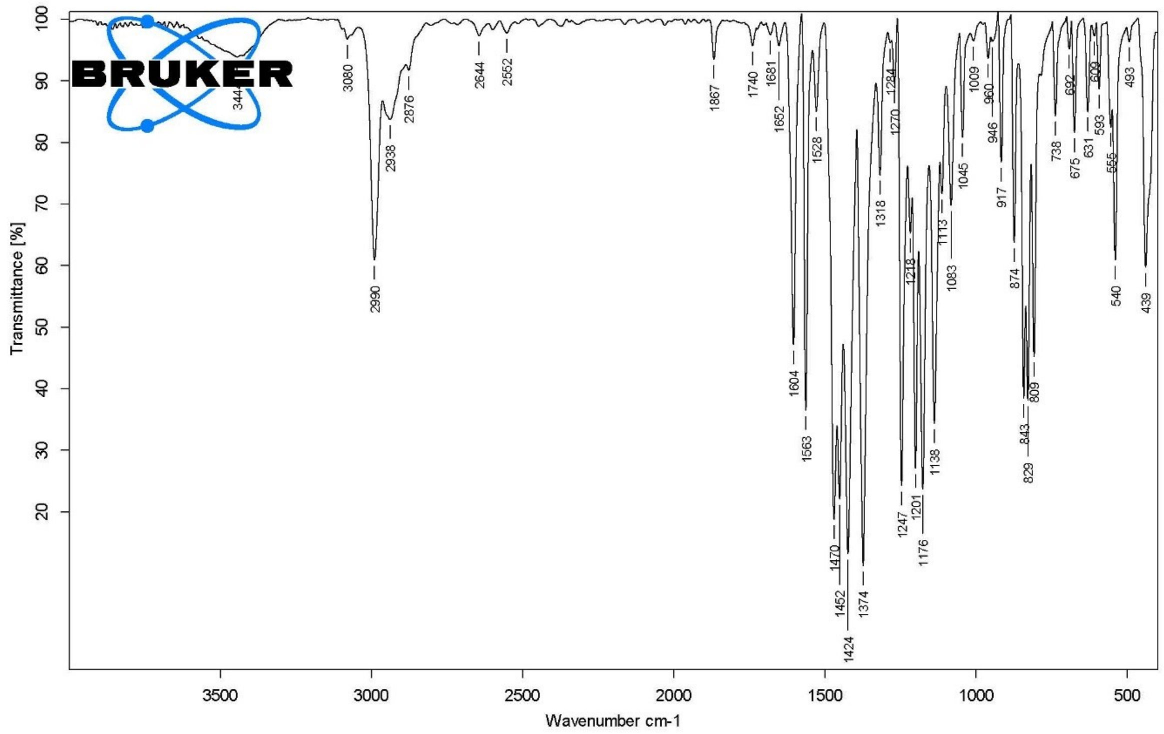

Figure S12. FT-IR spectrum of **4** in KBr.

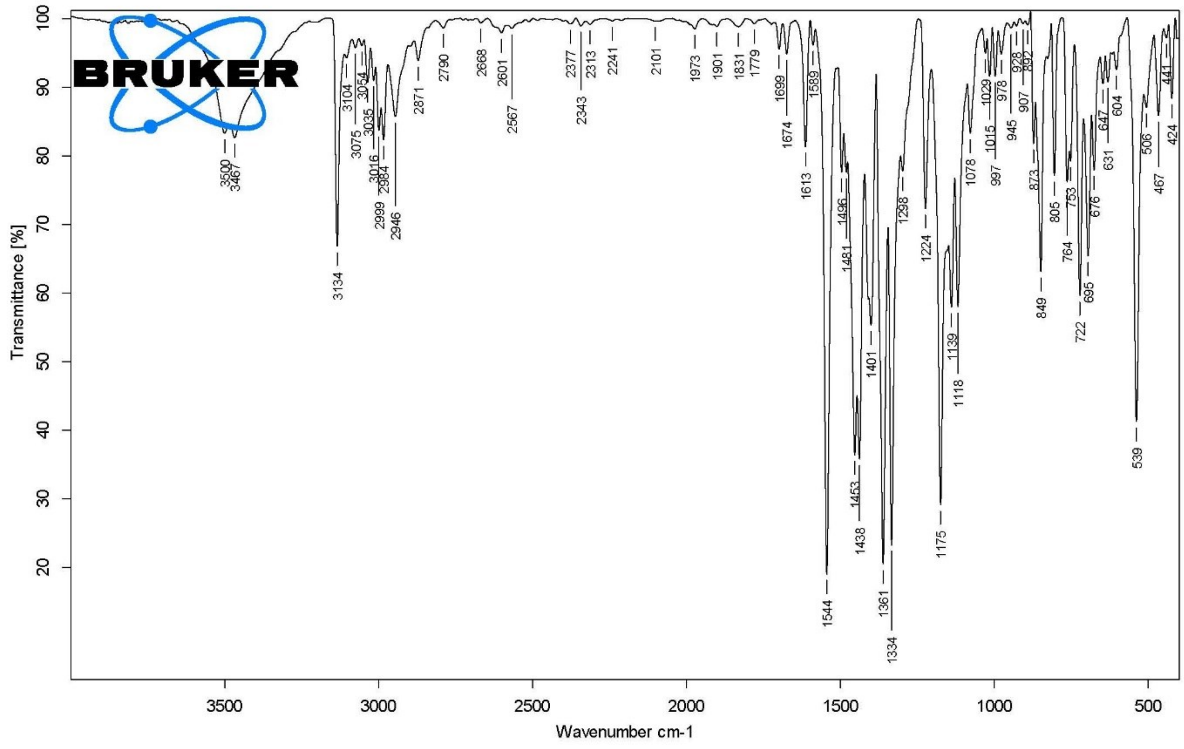

Figure S13. FT-IR spectrum of **7** in KBr.

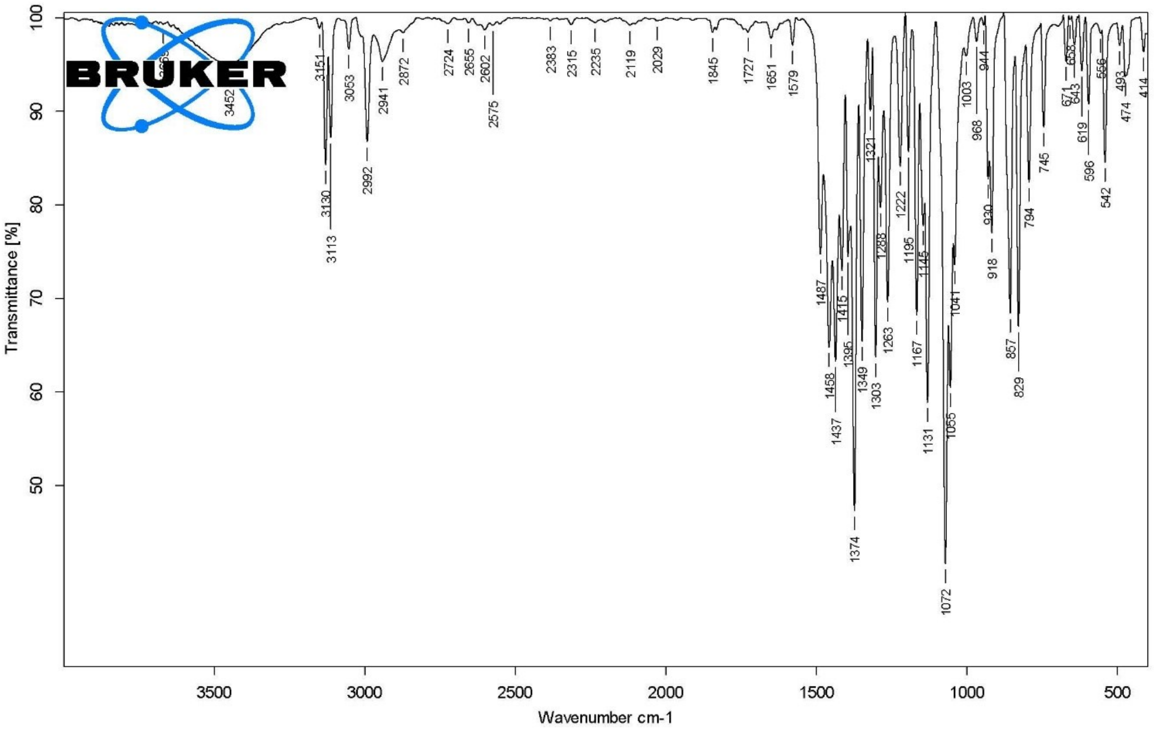

Figure S14. FT-IR spectrum of **8** in KBr.

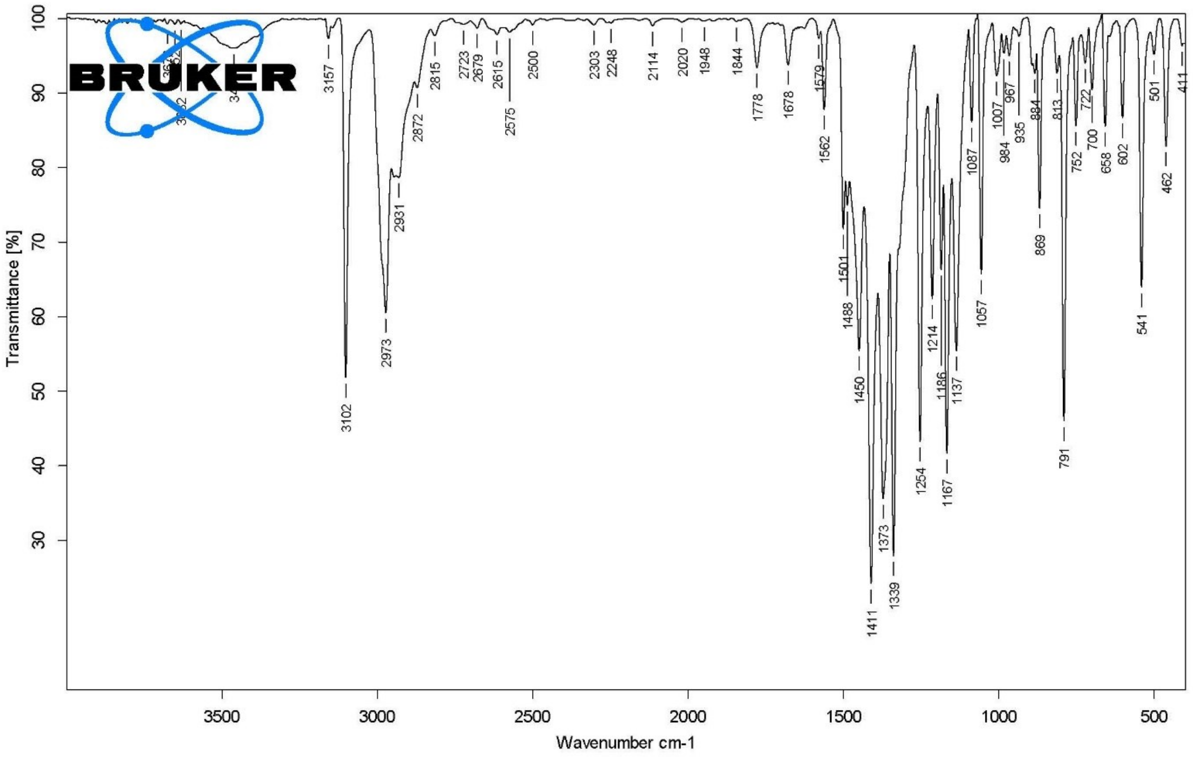

Figure S15. FT-IR spectrum of **9** in KBr.

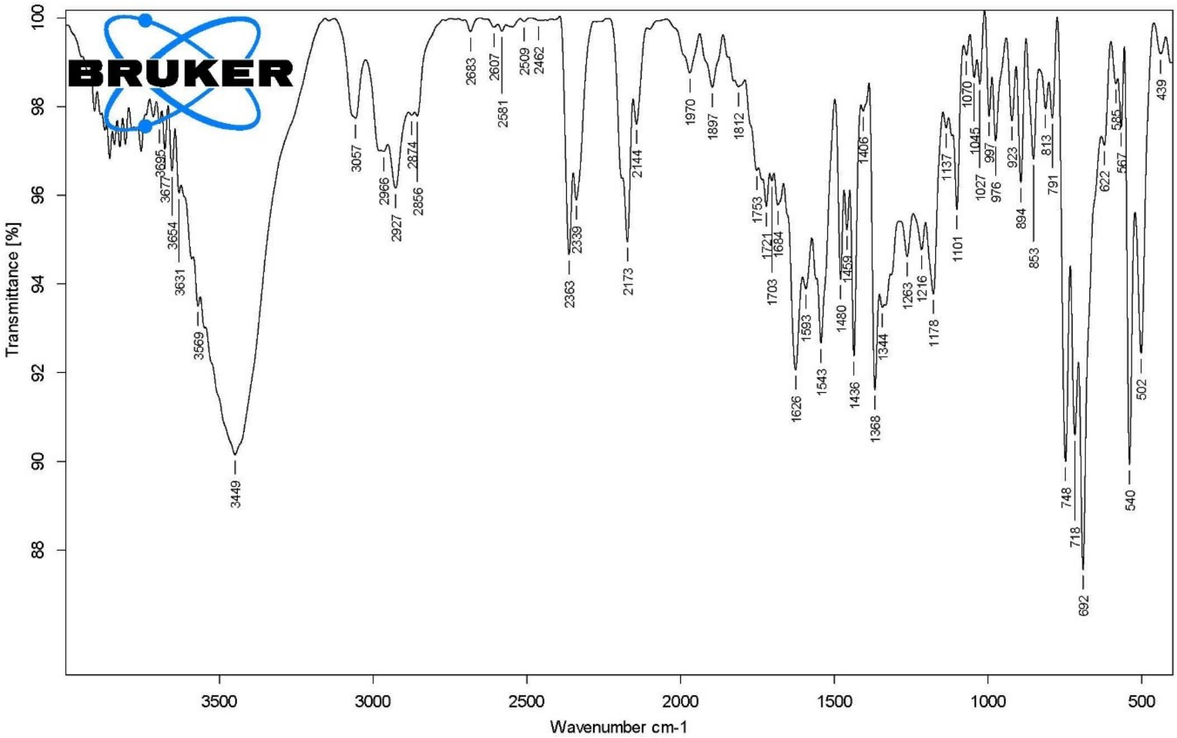

Figure S16. FT-IR spectrum of **11** in KBr.

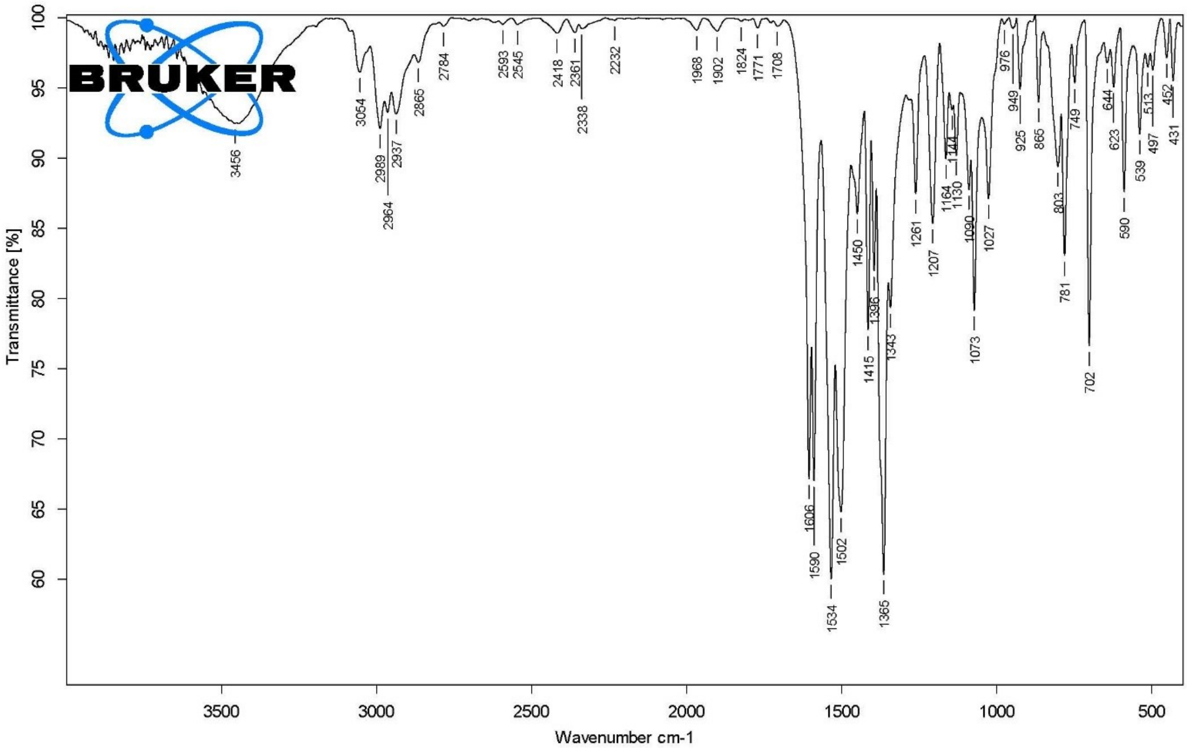

Figure S17. FT-IR spectrum of **13** in KBr.
